# The chromosome-scale genome of the raccoon dog: Insights into its evolutionary characteristics

**DOI:** 10.1016/j.isci.2022.105117

**Published:** 2022-09-15

**Authors:** Tianming Lan, Haimeng Li, Shangchen Yang, Minhui Shi, Lei Han, Sunil Kumar Sahu, Yaxian Lu, Jiangang Wang, Mengchao Zhou, Hui Liu, Junxuan Huang, Qing Wang, Yixin Zhu, Li Wang, Yanchun Xu, Chuyu Lin, Huan Liu, Zhijun Hou

**Affiliations:** 1 BGI Life Science Joint Research Center, Northeast Forestry University, Harbin 150040, China; 2 State Key Laboratory of Agricultural Genomics, BGI-Shenzhen, Shenzhen 518083, China; 3 College of Wildlife and Protected Area, Northeast Forestry University, Harbin 150040, China; 4 College of Life Sciences, University of Chinese Academy of Sciences, Beijing 100049, China; 5 College of Life Sciences, Zhejiang University, Hangzhou 310058, China; 6 Key Laboratory of Genetics and Germplasm Innovation of Tropical Special Forest Trees and Ornamental Plants (Ministry of Education), College of Forestry, Hainan University, Haikou 570228, China; 7 Shenzhen Zhong Nong Jing Yue Biotech Company Limited, Shenzhen 518120, China; 8 Guangdong Provincial Key Laboratory of Genome Read and Write, BGI-Shenzhen, Shenzhen 518120, China

**Keywords:** Animals, Genetics, Genomics, Zoology

## Abstract

The raccoon dog (*Nyctereutes procyonoides*) is an invasive canid species native to East Asia with several distinct characteristics. Here, we report a chromosome-scale genome of the raccoon dog with high contiguity, completeness, and accuracy. The intact taste receptor genes, expanded gene families, and positively selected genes related to digestion, absorption, foraging, and detoxification likely support the omnivory of raccoon dogs. Several positively selected genes and raccoon dog-specific mutations in *TDRD6* and *ZP3* genes may explain their high reproductivity. Enriched GO terms in energy metabolism and positively selected immune genes were speculated to be closely related to the diverse immune system of raccoon dogs. In addition, we found that several expanded gene families and positively selected genes related to lipid metabolism and insulin resistance may contribute to winter sleep of the raccoon dog. This high-quality genome provides a valuable resource for understanding the evolutionary characteristics of this species.

## Highlights

• Generated a high-quality chromosome-scale reference genome of the raccoon dog
• Identified and validated sex-linked genomic regions by resequencing 38 individuals
• Genomic basis for omnivory, high reproduction, immune system, and winter sleep
• Pre-introduction adaptation facilitated its successful invasion from Asia to Europe

## Graphical abstract

**Figure:**
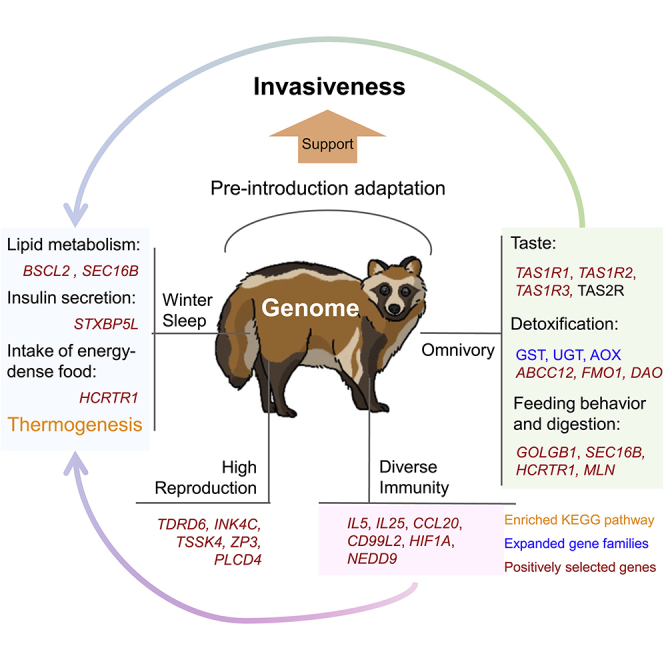

## Introduction

**Figure 1:**
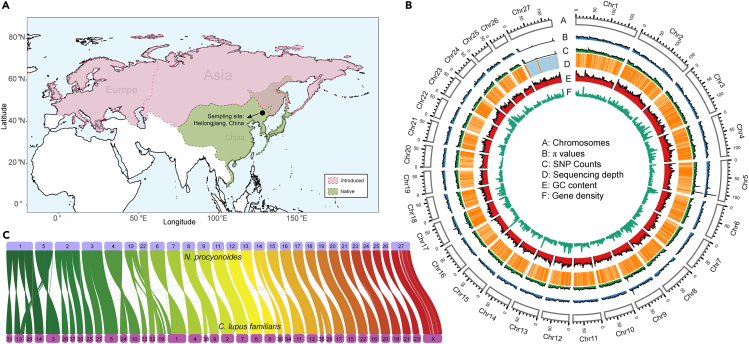
The distribution of native (green) and introduced/invasive (pink) areas of raccoon dogs, the landscape of the raccoon dog genome, and the chromosome-scale synteny analysis of the raccoon dog and domestic dog (A) The map describes the current distribution of raccoon dogs; here, we show both the native and introduced areas (https://www.cabi.org/isc/datasheet/72656). (B) The genomic landscape of the raccoon dog genome. A: The 27 chromosomes of the raccoon dog genome; B: population level genetic diversity (π) calculated by a 500 kb window; C: SNP density across the genome (500 kb window); D: sequencing depth (X) calculated by 500 kb window; E: GC content (%); F: gene density calculated by 500 kb window. (C) The chromosome-scale synteny analysis between the raccoon dog genome and the domestic dog genome, which was visualized using RectChr v1.27 (https://github.com/BGI-shenzhen/RectChr).

The raccoon dog (*Nyctereutes procyonoides*) is the only species in the genus Nyctereutes. It is a medium-sized canid that prefers various habitats such as gardens, marshlands, river valleys, and damp forests with rich undergrowth (Kauhala and Kowalczyk, 2011). The native distribution area of raccoon dogs includes China, Korea, North Vietnam, Japan, Mongolia, and East Siberia (Pitra et al., 2009; Ward and Wurster-Hill, 1990) (Figure 1A). The range of this species has continuously expanded over the last 100 years, and currently covers most of Eurasia, making the raccoon dog a prominent invasive species in Europe and posing a great risk to local ecosystems and public health.

Globally, there are six subspecies (Ellerman and Morrison-Scott, 1951; Hong et al., 2018; Ward and Wurster-Hill, 1990), including *N. procyonoides* ussuriensis (originally found in eastern China and southeastern Siberia), *N. procyonoides procyonoides* (northern Indochina and China)*, N. procyonoides orestes* (southwestern China)*, N. procyonoides koreensis* (Korean Peninsula)*, N. procyonoides viverrinus* (Japan, except Hokkaido), and *N. procyonoides albus* (Hokkaido, Japan). The *N. procyonoides viverrinus* and *N. procyonoides albus* subspecies were suggested to be recognized as distinct species considering the large difference in the number of chromosomes between Japanese raccoon dogs (2n = 38 + 3–4 B) and other raccoon dogs (2n = 54 + 2–3 B) (Nie et al., 2003).

There are several distinct evolutionary characteristics in raccoon dogs. This species is reported to have a high reproductive rate with a relatively young age of sexual maturity and large average litter size (8–10 individuals) (Helle and Kauhala, 1995; Kauhala, 1996; Kauhala and Kowalczyk, 2011; Kowalczyk et al., 2009). The raccoon dog is a typical omnivore with a diverse diet that includes food ranging from fruits to insects, frogs, birds, and small mammals (Drygala et al., 2013; Kauhala and Kowalczyk, 2011). This helps raccoon dogs shift their diets according to the different habitats and seasons, making the raccoon dog a species with high plasticity. Another unique habit of raccoon dogs among canids is winter sleep under harsh conditions, which can protect raccoon dogs from food deprivation and cold weather (Asikainen et al., 2004). Before winter sleep, raccoon dogs fatten themselves and their body weight will be correspondingly increased (Nieminen et al., 2002). Unlike typical hibernation, the body temperature of the raccoon dog is close to normal (Asikainen et al., 2004). However, little is known about the possible genetic basis of these biological characteristics.

In addition, raccoon dogs are regarded as reservoir hosts for many pathogens, which poses a great threat to public health, because related diseases can also be transmitted to humans and other animals (Kauhala and Kowalczyk, 2011; Kjaer et al., 2021; Laurimaa et al., 2016; Sutor et al., 2014). The raccoon dog is a very important vector of rabies, as a large percentage of rabies cases are found in raccoon dogs, even higher than that in red foxex (Kauhala and Kowalczyk, 2011). In addition, the raccoon dogs have also been found to be hosts of SARS-CoV (Guan et al., 2003), H5N1 virus (Qi et al., 2009), canine distemper virus (Aoyagi et al., 2000), porcine circoviruses (Song et al., 2019), and Amdoparvovirus (Shao et al., 2014). The raccoon dog is also a reservoir species of many parasites, including *Alaria alata*, *Echinococcu multilocularis*, *Sarcoptes scabiei*, and *Trichinella* spp. (Kauhala and Kowalczyk, 2011). Other tick-borne pathogens, such as *Anaplasma phagocytophilum*, are also detected in raccoon dogs (Kjaer et al., 2021). We anticipated that the immune system of raccoon dogs is diverse.

A high-quality genome is a valuable genetic resource to explore the possible genetic basis for the biological features of a species. Chueca et al. recently reported a genome assembly of the raccoon dog, but with a female individual (Chueca et al., 2021). In this study, we assembled a chromosome-scale genome of raccoon dogs by using a male individual collected from Heilongjiang, China. We explored the possible genetic basis of the biological characteristics of raccoon dogs, including immunity, reproduction, omnivory, winter sleep, and invasiveness. The findings from this study will provide a useful and valuable genomic resource for future research on the evolution, ecology, and management of wild populations of this species.

## Results

### Improved genome assembly

A total of 385.94 Gb long reads generated by the Pacific Biosciences (PacBio) platform, 175.52 Gb whole-genome sequencing (WGS) short reads and 203.52 Gb of Hi-C reads were used for the chromosome-scale genome assembly (Table 1). We first assembled one primary genome with a contig N50 of 23.99 Mb by using error-corrected PacBio subreads. Then, this primary genome was polished by PacBio subreads and WGS short reads. Finally, Hi-C reads were used for concatenating primary contigs into chromosome-scale assembly. The raccoon dog genome contains 27 pairs of chromosomes (2n = 54), including 26 pairs of autosomes and one pair of sex chromosomes (Nie et al., 2003). The size of the final assembled nuclear genome was 2.38 Gb, and the genome consisted of 218 scaffolds after redundancy removal, with 2.32 Gb assigned to 27 chromosomes (Table S1, Figure S1, and Figure 1B). The scaffold N50 of this chromosome-scale assembly was 41.87 Mb and the GC content of this genome was 41.33%, which is very similar to that of the domestic dog (CanFam3.1, GCA_000002285.2) and red fox (VulVul2.2, GCA_003160815.1) genomes (Table 1).

**Table 1:** Statistics for the sequencing data, genome assembly, and annotation of the raccoon dog genome

Item	Category	Number
Sequencing data	PacBio (Gb)	385.94
	WGS (Gb)	175.52
	Hi-C (Gb)	203.52
	RNA-seq (5 organs) (Gb)	101.70
Assembly	Estimated genome size (Gb)	3.21
	Assembled genome size (Gb)	2.38
	Contig N50 (Mb)	41.87
	Scaffold N50 (Mb)	83.70
	Longest scaffold (Mb)	177.96
Annotation	GC content (%)	41.33
	Repeat sequences (%)	35.11
	Number of protein-coding genes	20,000
	Number of functionally annotated genes	19,973

To evaluate the completeness of the genome, we first performed the Benchmarking Universal Single-Copy Orthologs (BUSCO) (Simão et al., 2015) analysis by using the mammalia_odb10 database. We found that 96.4% of the 9,226 BUSCO genes were complete in the genome, and only 2.8% and 0.8% were identified as missing and fragmented, respectively (Table S2). To compare with the previously published genome (GCA_905146905.1, Rac 1.0 hereafter), we also used the laurasiatheria_odb10 database to perform the BUSCO analysis (Table S2). The BUSCO score of our genome was slightly higher than that of the Rac 1.0 genome. In addition, 96.28% of the transcript data (kidney) and 99.26% of the WGS short reads were mapped onto our final assembly (Table S3). We also downloaded the RNA-seq data of the Rac 1.0 genome and mapped it against its own genome and the genome we assembled. We found that the mapping rate on our genome (96.24%) was higher than that on the Rac 1.0 genome (85.75%). The above assessments showed that our assembly represents a more complete genome with high quality and contiguity.

To further evaluate the accuracy of our assembly at the chromosome level and detect the fusion and fission events, we performed an interspecies synteny analysis between our assembled genome and the domestic dog genome (CanFam3.1, GCA_000002285.2). Overall, we found high collinearity between our assembled genome and the domestic dog genome with clear one-to-one syntenic blocks (Figure 1C). We also found nine fusion and three fission events in the synteny result. For example, Chr1 is the largest chromosome of the raccoon dog, which was found to be a fusion of Chr13, Chr14, Chr29, and Chr31 of the domestic dog genome. Chr1, Chr13, and Chr19 of the domestic dog genome were split into two chromosomes in the raccoon dog genome. All these fission and fusion events were surprisingly consistent with the findings of a previous karyotypic study (Becker et al., 2011), indicating the accuracy of our assembled genome at the chromosome level.

### Genome annotation

We first identified 835.98 Mb of repetitive elements in our assembled genome, representing 35.11% of the total genome size. These repeat elements included LINEs (22.71%), LTRs (12.00%), DNA elements (2.40%), SINEs (1.23%), and other repeats (0.34%) (Tables S4–S6). Our genome’s total repeat length was found to be somewhat larger than that of the Rac 1.0 genome (34.04%), demonstrating the superiority of our genome’s longer contigs in assembling highly repeated regions.

We sequenced 101.70 Gb of RNA-seq data and generated 175,521 full-length transcripts to support the gene annotation of our genome. We finally identified 20,000 protein-coding genes with high confidence by combining high-quality transcript mapping, homology-based protein alignment, and *ab initio* predictions. The average exon length, intron length, and gene length were 176.40 bp, 4481.96 bp, and 38.22 kb, respectively (Figure S6). Among our predicted genes, 14,913 (74.57%) were supported by transcript data. The BUSCO score of our predicted gene set was evaluated as 92.7% with the mammalia_odb10 database, showing high completeness (Table S2). Finally, 19,973 (99.87%) genes were functionally annotated in at least one of the five databases we used (Table S7 and Figure S7). In addition, 781 rRNAs, 995 miRNAs, 2,280 snRNAs, and 146,963 tRNAs were predicted in our genome (Table S8).

### Identification of sex-linked regions

**Figure 2:**
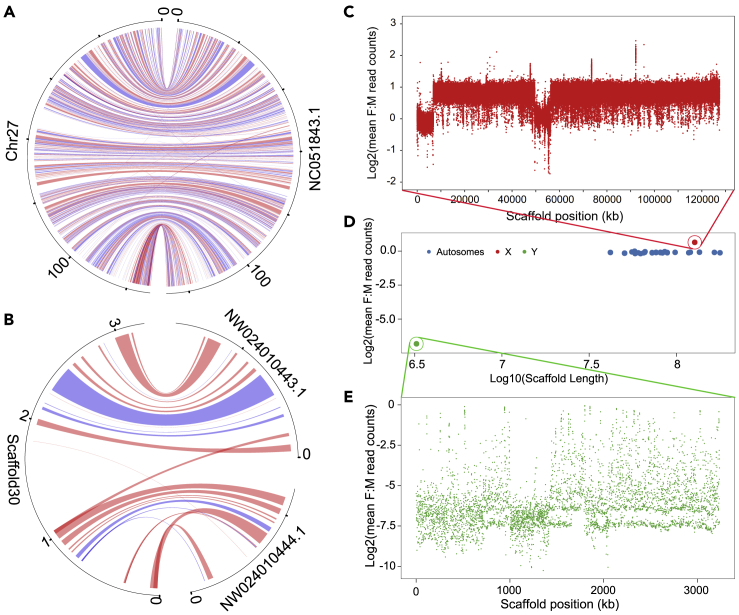
Identification of the X chromosome and Y-linked scaffold (A) Synteny analysis of genes on the X chromosome between raccoon dog and domestic dog. Red lines indicate the genes in the dog genome mapped to the positive strand of the raccoon dog genome, and blue lines indicate the genes in the dog genome mapped to the negative strand of the raccoon dog genome. (B) Synteny analysis of genes on the Y chromosome between raccoon dog and domestic dog. The red line and blue line are the same as those in (A). (C) The ratio of sequencing depth between female and male individuals in a 500 bp window across the X chromosome. (D) The ratio of the sequencing depth of each chromosome-scale scaffold between female and male individuals. The red dot represents the X chromosome, the green dot represents the Y-linked scaffold, and the blue dots are scaffolds from autosomes. The expected ratio is 1:1, the X chromosome is expected to have a higher ratio and the Y chromosome is expected to have a lower ratio. (E) The ratio of sequencing depth between female and male individuals in a 500 bp window across the Y-linked scaffold.

Considering that the Rac 1.0 genome was assembled using a female individual, we determined both X- and Y-linked scaffolds with multiple lines of evidence. We first ranked the average sequencing coverage of all 218 scaffolds (Table S9) and found that Chr27 and Scaffold30 presented sequencing coverages with nearly half of the whole genome level (Figure S8). We then regarded these two genome regions as derived from sex-linked chromosomes. A corresponding low level of genetic diversity was also found in these scaffolds, which further supported our identification of sex chromosomes (Table S10). To further separate the Y- and X-linked regions, we performed synteny analysis between our primarily identified sex-linked regions and the X/Y chromosomes of the domestic dog genome (Figures 2A and 2B). We found 797 and 25 protein-coding genes in the domestic dog X and Y chromosomes, respectively, that were successfully mapped onto Chr27 and Scaffold30 (Table S11) in the raccoon dog genome. The assembled X and Y chromosomes of the raccoon dog presented high collinearity with the X and Y chromosomes of the domestic dog. We therefore concluded that Chr27 (127.44 Mb) and Scaffold30 (3.24 Mb) were the X chromosome and Y-linked scaffold, respectively.

To further validate our identification, we mapped the whole genome resequencing data from 38 individuals of known sex (5 females and 33 males) to our assembled genome (Table S12). As we expected, the sequencing depth of Chr27 and Scaffold30 in the male individuals was nearly half that of the autosomes (Figures 2C–2E). For the female individuals, however, the depths of Chr27 were nearly the same as those of the autosomes (Figures 2C–2E). These results further supported that our identification of the sex-linked regions was accurate. This is the first time we identified X and Y sex-linked genomic regions in a raccoon dog reference genome, which will be a valuable resource for future studies.

### Expansion of gene families in the raccoon dog genome

To explore the genomic adaptations of raccoon dogs, we performed a comprehensive comparative genomic analysis with 17 other Carnivora and mammalian species (Table S13). We focused on biological characteristics such as reproduction, immunity, omnivory, and winter sleep, which are prerequisites for raccoon dogs to spread and adapt to habitats. To better understand the possible genetic basis of the omnivorous diet, we included herbivores, omnivores, and carnivores.

**Figure 3:**
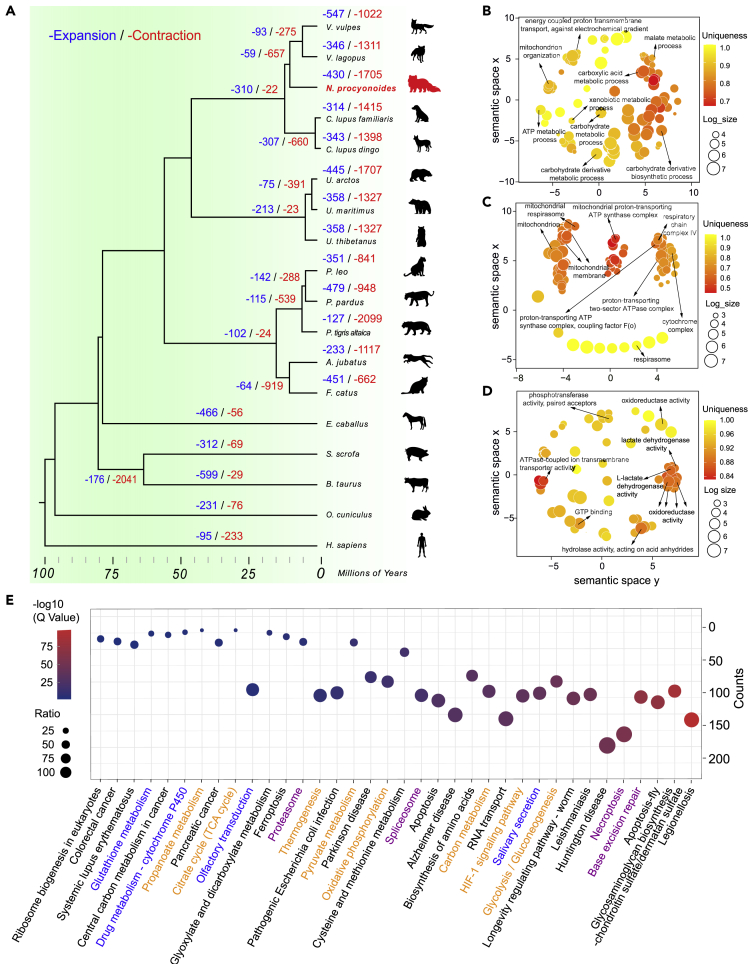
Comparative genomics analysis and enrichment analysis of expanded gene families in the raccoon dog genome (A) The phylogenetic relationship of 18 species and the estimated divergence time. Numbers on the branch of the phylogenetic tree represent the significantly expanded (blue) and contracted (red) gene families. (B–D) Clusters of significantly overrepresented GO items for biological process (B), cellular component (C), and molecular function (D) by REVIGO for expanded gene families in the raccoon dog genome. Semantic similar GO terms clustered together. (E) Significantly enriched KEGG pathways in the raccoon dog genome compared with the other 17 species. Blue: pathways related to the omnivorous diet. Orange: pathways related to energy metabolism. Purple: pathways related to immunity.

We found 430 expanded gene families in the raccoon dog genome compared with the common ancestor of the raccoon dog, red fox, and arctic fox (Figure 3A). GO enrichment analysis of these expanded gene families showed that a large proportion of significantly enriched GO terms were closely related to energy metabolism, including carbohydrate metabolic process (GO:0,005,975, p = 0.0025), ATP metabolic process (GO:0,046,034, p = 2.92 × 10^−26^), ATP synthesis coupled proton transport (GO:0,015,986, p = 5.55 × 10^−46^), energy coupled proton transport, down electrochemical gradient (GO:0,015,985, p = 5.55 × 10^−46^), and malate metabolic process (GO:0,006,108, p = 5.47 × 10^−8^) (Figures 3B–3D, Table S14). We also found an enriched GO term that was related to detoxification (drug metabolic process, GO:0017144, p = 3.77 × 10^−23^) and lipid metabolism (GO:0055102, p = 0.0034). In the 37 significantly enriched KEGG pathways (Table S15, Figure 3E), we found that eight pathways were closely related to energy metabolism, including the citrate cycle (map00020, p = 1.76 × 10^−4^), glycolysis (map00010, p = 1.33 × 10^−44^), oxidative phosphorylation (map00190, p = 7.21 × 10^−18^), pyruvate metabolism (map00620, p = 1.95 × 10^−12^), carbon metabolism (map01200, p = 8.95 × 10^−34^), thermogenesis (map04714, p = 8.36E-10), HIF-1 signaling (map04066, p = 7.96 × 10^−38^), and propanoate metabolism (map00640, p = 0.0014) pathways. Four significantly enriched pathways were found to be immune-related, including base excision repair (map03410, p = 1.22 × 10^−72^), necroptosis (map04217, p = 2.47 × 10^−56^), spliceosome (map03040, p = 3.65E-20), and proteasome (map03050, p = 1.13 × 10^−6^). We also found some enriched pathways that were related to detoxification (drug metabolism-cytochrome P450, map00982, p = 0.0035 and glutathione metabolism, map00480, p = 0.0076) and the biosynthesis of amino acids (map01230, p = 1.54 × 10^−32^). In addition, olfactory transduction (map04740, p = 5.79 × 10^−5^) and salivary secretion (map04970, p = 3.52 × 10^−39^) were also significantly enriched pathways in the raccoon dog genome (Figure 3E and Table S15).

### Evolution of genes associated with omnivory, reproduction, immunity, and winter sleep

**Figure 4:**
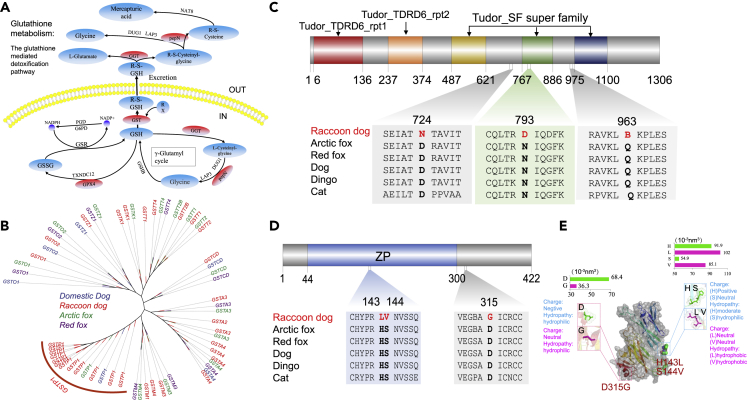
The possible genetic basis for detoxification and high reproduction in the raccoon dog (A) The glutathione-mediated detoxification pathway. The ellipse in red represents enzymes encoded by expanded gene families. (B) The phylogenetic tree of the GST gene family was constructed by the maximum likelihood method. Blue: Domestic dog; Red: Raccoon dog; Green: Arctic fox; Purple: Red fox. The GSTP1 genes in the raccoon dog genome are obviously expanded. (C) Raccoon dog-specific amino acid changes in the *TDRD6* gene. N793D was found in the Tudor_SF superfamily. (D) Raccoon dog-specific amino acid changes in the *ZP3* gene. Two substitutions were found to be in the ZP domain region. (E) Three-dimensional view of the ZP3 protein, highlighting raccoon dog-specific amino acid changes. The zoomed in pink amino acids are the raccoon dog-specific amino acids and the green amino acids are the those predicted from the dog. The bar plot shows the residual volume of amino acids.

Raccoon dogs are opportunistic omnivores, as they mainly consume easily found food. Their diet comprises small mammals, insects, fruit, plant seeds and leaves, and carrion comprise (Hirasawa et al., 2006; Mulder, 2012). Toxic secondary metabolites usually exist in plants (Xia et al., 2021) and toxic byproducts are also widely distributed in rotting food (Blumstein et al., 2017). In addition, poisonous substances such as plant alkaloids are typically bitter in taste (Li and Zhang, 2014). Therefore, we focused on detoxification and taste-receptor-related gene families. In the expanded gene families, we found some typical detoxification gene families, including glutathione S-transferase (Hayes and Pulford, 1995; Sayyab et al.), protein tyrosine phosphatase (UGT) (Bock, 2016), and aldehyde oxidase (Chang et al., 2010; Guo et al.). Interestingly, we found that the GST gene family was expanded in the raccoon dog genome in all groups, including omnivores, herbivores, and carnivores (Figures 4A and 4B). Sweet taste, umami taste, and bitter taste are mediated by the taste receptors TAS1R2/TAS1R3, TAS1R1/TAS1R3, and TAS2Rs, respectively. In this study, we found that *TAS1R1*, *TAS1R2*, *TAS1R3*, and several TAS2R genes were intact and functional in the raccoon dog genome (Table S16). Selection analysis showed that three detoxification-related genes, *ABCC12, FM O 1*, and *DAO*, were under positive selection. Feeding behavior and digestion-related genes (*GOLGB1*, *SEC16B*, and *MLN*) were also found to be under positive selection (Table S17).

Although we did not find significantly enriched GO or KEGG pathways that were directly related to reproduction in expanded gene families, five reproductive genes, *TDRD6*, *CDKN2C*, *ZP3*, *PLCD4*, and *TSSK4*, were found to be positively selected in the raccoon dog genome. These genes are closely related to spermatogenesis and acrosomal reactions. In the *TDRD6* and *ZP3* genes, we found that each gene contains three raccoon dog-specific amino acid changes (Figures 4C and 4D). The mutation N793D is located in the Tudor superfamily domain of the *TDRD6* gene, and the S144V mutation was found in the ZP domain region of the *ZP3* gene. We then predicted the effects of these mutations on protein function by using the Protein Variation Effect Analyzer (Provean) web server (Choi and Chan, 2015). Five of the six mutations in the *TDRD6* and *ZP3* genes were predicted to be neutral by Provean, and we did not find damaging effects in the amino acid changes in the domain regions (Table S18). We further predicted the three-dimensional structure of the ZP3 protein. Although these amino acid substitutions did not change the main structure of this protein, the physicochemical properties of the amino acids changed, which may contribute to the high reproduction of the raccoon dog (Figure 4E).

By comparison with the above mentioned 17 species, we screened 206 genes that were under positive selection. Thirty genes, accounting for 14.56% of the total positively selected genes (PSGs), were immune-related, including *IL5*, *IL25*, *CCL20*, *CD99L2*, *HIF1A*, and *NEDD9* (Table S19). We also performed a comparative genomic analysis on all 30 genes with other canines. Thirty-five (35) amino acid changes harbored in 18 genes were found to be raccoon dog-specific changes, and 21 mutations were found to be located in functional domain regions (Table S20, Figure S9). To test the reliability of these mutations, we checked the read mapping of these mutations and found that all mutations were real and well supported by read mapping. Further inspection found that 32 of these mutations were fixed in the population (Table S21). We also found that 10 tumor suppressor genes (*RERG*, *BRCA1*, *FETUB*, *RNF20*, *MY O 18B*, *RBM5*, *BMP3*, *LACTB*, *UNC5A*, and *PCK1*) (Table S22) and 6 energy metabolism-related genes (*CDKAL1*, *TRIM63*, *GALNT13*, *PRKAG3*, *RPUSD4*, and *MRPL19*) were under positive selection (Table S23).

To further explore the possible genetic basis of winter sleep in raccoon dogs, we performed selection analysis by comparing raccoon dogs with other 14 other non-hibernating animals (Table S24). In total, we found that 194 genes were under positive selection (Table S25). As expected, we found 21 immune-related genes (Table S26) and five energy metabolism-related genes (Table S27). Genes with feeding behaviors were also found to be under positive selection (*HCRTR1*, *GOLGB1*, and *MLN*) (Table S28). In particular, we found three genes related to insulin secretion (*STXBP5L*) and lipid metabolism (*SEC16B* and *BSCL2*) (Table S28).

### Population history and genetic diversity

**Figure 5:**
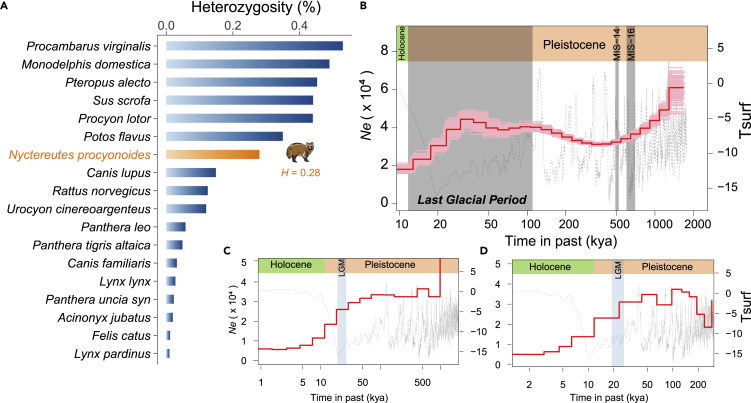
Population history and genome-wide heterozygosity (A) The genomic heterozygosity of the raccoon dog and 17 other species. The genomic heterozygosity of 17 species was collected from published data. (B) The population history of the raccoon dog inferred by PSMC with 100 bootstraps. The red line represents the estimated effective population size (*N*e), and the 100 thin red lines represent the PSMC estimates of 100 randomly resampled from the original sequence. Tsurf: atmospheric surface air temperature relative to the present. The mutation rate (μ) and generation interval (g) used here were 1.0 × 10^−8^ and 3 years, respectively. (C) The recent population history of the raccoon dog inferred by MSMC2 with four individuals. LGM: Last Glacial Maximum. We used the same μ and g as used in the PSMC analysis. (D) The recent population history of the raccoon dog inferred by SMC++ with 38 individuals. We used the same μ and g as used in the PSMC analysis.

Although the genetic diversity of raccoon dogs has been explored by using mtDNA and microsatellite loci (Hong et al., 2018; Slaska and Grzybowska-Szatkowska, 2011; Slaska et al., 2010), this is the first study reporting genetic diversity evaluation at the whole-genome scale. To facilitate comparison with other species, we collected the heterozygosity (*H*) data of 17 other species, including the Felidae, Canidae, and some other invasive species (Table S29). The genome-wide *H* of the raccoon dog was identified as 0.28 (Figure 5A). This *H* is lower than some other invasive species; however, it is obviously higher than many Felidae and Canidae species listed in this study (Figure 5A and Table S29).

We reconstructed the history of effective population size (*Ne*) of the raccoon dog from ∼1.6 million years ago (Mya) to nearly 1,000 years ago (kya), by combining the PSMC (Li and Durbin, 2011), MSMC2 (Schiffels and Durbin, 2014), and SMC++ (Terhorst et al., 2017) methods. The PSMC result showed that the *Ne* gradually declined from ∼1.6 Mya to ∼320 kya and then experienced an expansion from ∼320 kya to ∼100 kya. The *Ne* was relatively stable between ∼100 kya and 50 kya, and the population declined again to 10 kya (Figure 5B). The PSMC usually shows poor performance for inferring recent population history (Li and Durbin, 2011). We therefore used MSMC2 to infer a more recent population history, and the results showed that *Ne* presented a continuous decline from 50 kya to 1.0 kya without any rebounding (Figure 5C). Since the switch error rate induced by phasing influences the accuracy of MSMC2, we further validated the results from MSMC2 by using SMC++, which is independent of the phasing process. As we expected, the trend in *Ne* inferred by SMC++ was extremely similar to that inferred by MSMC2 (Figure 5D).

## Discussion

### Improved genome assembly and annotation

A chromosome-scale genome assembly with high completeness, accuracy, and contiguity can provide a solid foundation for genetic research (Rhie et al., 2021). Although a draft genome of the raccoon dog has been recently reported (Chueca et al., 2021), the authors used a female individual for genome assembly, which lacked data for the Y chromosome. The raccoon dog genome assembly needs to be further improved. Here, we report a comprehensively improved chromosome-scale genome by combining PacBio and Hi-C technologies for this species. This genome showed 1.19-fold and 1.55-fold improvements in N50 values of the contig and scaffold over those of the Rac 1.0 genome (Chueca et al., 2021), respectively, indicating substantial improvement in this chromosome-scale genome assembly in terms of contiguity. The karyotypic study of the raccoon dog showed 26 pairs of autosomes, one pair of allosomes, and 2 to 3 B chromosomes (Nie et al., 2003). Here, we identified 27 chromosomes by Hi-C scaffolding, which was consistent with the karyotyping result. Surprisingly, all fission and fusion events between the domestic dog and raccoon dog genomes were highly consistent with those found in a previously published karyotypic study (Nie et al., 2003), indicating the high accuracy of this assembly at the chromosome level. In addition, we identified a 127.44 Mb X chromosome and 3.24 Mb Y-linked region with multiple lines of evidence. This is the first time we identified a genome-wide Y-linked region in the raccoon dog. The genome size estimated in this study was ∼3.21 Gb (Figure S10), which is similar to that estimated by flow cytometry (Chueca et al., 2021), indicating the existence of B chromosomes. However, we failed to assemble the B chromosomes probably due to clusters of telomeric sequences along the whole B chromosome (Bugno-Poniewierska et al., 2014). For the gene annotation, although the gene number we identified is much less than that in the Rac 1.0 genome, the number we annotated is more comparable with those of other Canidae species (Kukekova et al., 2018; Peng et al., 2021). Altogether, we presented a much-improved raccoon dog genome, which will be a valuable resource for future studies.

### Genetic basis for the omnivorous diet of the raccoon dog

The raccoon dog is a typical omnivorous canid species. We found that genes encoding receptors that mediate sweet, bitter, and umami tastes all exist in the raccoon dog genome and are structurally intact. As in humans, the most typical representatives of omnivores, these functional taste receptor genes provide the basic genetic basis for the omnivorous diet (Gravina et al., 2013). The sweet and umami taste receptors help the raccoon dog to forage fruits and meat, and the bitter taste receptor plays an important role in recognizing poisonous substances such as plant alkaloids that are typically bitter in taste (Li and Zhang, 2014). However, many toxic secondary metabolites in plants (Xia et al., 2021) and toxic byproducts in rotting food (Blumstein et al., 2017) cannot be tasted. GST and CYP450 are reported to be the main enzymes that catalyze the phase I and phase II reactions of many toxic substances in organisms (Zhao et al., 2020). The expanded gene families in the raccoon dog were enriched in one detoxification-related GO term (drug metabolic process) and two KEGG pathways (drug metabolism-cytochrome P450 and glutathione metabolism). The cytochromes P450 (CYP) superfamily is a large enzymatic protein family in both animals and plants that participates in multiple and critical physiological processes (Danielson, 2002; Xu et al., 2015). One of the most important functions of the CYP450 superfamily is the degradation of toxic substances, which has been reported in many species (Gui et al., 2020; Li et al., 2018). Glutathione (GSH) plays an important role in detoxification by forming GSH-toxin conjugates (either with xenobiotic or endogenous electrophilic compounds) (DeLeve and Kaplowitz, 1991; Ketterer et al., 1983) (Figure 4A). Although some conjugations can occur spontaneously, conjugations of GSH to a wide variety of toxic substances are catalyzed by GST, a powerful detoxification enzyme (DeLeve and Kaplowitz, 1991). Interestingly, we found that the GST gene family was significantly expanded in the raccoon dog genome compared with omnivorous, herbivorous, and carnivorous animals. GST can affect the activity of many antioxidant enzymes to help remove contaminants and reactive oxygen species (Yao et al., 2020). In addition, we also found that the *ABCC12* gene was under positive selection compared with other canids. The *ABCC12* gene is a member of the ABC gene family, and the proteins encoded by this gene family play a role in detoxification (Dermauw and Van Leeuwen, 2014). Although the exact function of *ABCC12* is unknown, this gene is a member of the ABCC subfamily, which belongs to the group of multidrug resistance protein (MRPs) (Ono et al., 2007; Whitlock and Leslie, 2020), which play important roles in the function of GST pumps (Muller et al., 1996). Taken together, from these results, we inferred that GST, which is involved in glutathione metabolism, plays an important role in physiological processes associated with detoxification, perhaps even being more important than the CYP 450 superfamily in raccoon dogs. We also found two other detoxification-related genes that were under positive selection, *FM O 1* and *DAO*. FMO genes can be found across the whole animal kingdom, in which they play roles in the detoxification of xenobiotics by catalyzing the conversion of heteroatom-containing chemicals to excretable metabolites (Sehlmeyer et al., 2010). The *DAO* gene encodes diamine oxidase, which also plays an important role in dietary histamine detoxification to reduce the uptake of histamine by enterocytes (Yoshikawa et al., 2019). All these detoxification-related genes, gene families, and pathways are possible candidates for the genetic basis of the raccoon dog’s broad diet.

### High reproduction rate from a genetic perspective

The raccoon dog has a high reproductive rate with an average litter size of 8–10 individuals (Helle and Kauhala, 1995; Kauhala, 1996; Kauhala and Kowalczyk, 2011; Kowalczyk et al., 2009), which is larger than that of the red fox, its close relative (Kauhala, 1996). Although we did not find significantly enriched GO and KEGG pathways that are directly related to reproduction in the raccoon dog genome, we still found several reproduction-associated genes that were under positive selection. The *TDRD6* (Vasileva et al., 2009), *INK4C* (Zindy et al., 2001), and *TSSK4* (Wang et al., 2015) genes were reported to be involved in spermatogenesis. The development of spermatids from round to elongated morphology will be abrogated in mice with *TDRD6* gene knockout (Vasileva et al., 2009). *INK4C* and *INK4D* are two essential genes for male fertility and collaborate in regulating spermatogenesis. The deletion of *INK4C* and *INK4D* in mice results in male infertility (Zindy et al., 2001). Mutation in the *TSSK4* gene was also found to be harmful to spermatogenesis in infertile Chinese men (Su et al., 2008). The *ZP3* (Wassarman, 1999) and *PLCD4* (Fukami et al., 2001) genes play key roles in the acrosome reaction. The acrosome reaction is the fusion process of sperm and egg cells, and ZP3 is considered the sperm receptor of the oocyte and triggers the acrosome reaction (Jungnickel et al., 2007); *PLCD4* is responsible for mediating this zona pellucida-induced acrosome reaction (Fukami et al., 2001)*.* We speculated that these genes may help to maintain high reproduction in raccoon dogs, especially for males. The raccoon dog-specific amino acid changes were also found in the *ZP3* and *TDRD6* genes (Figures 4C and 4D), which may also play positive roles in the function of spermatogenesis and acrosome reaction. However, experimental analysis is necessary for further validation. In addition, many of these genes were pleiotropic (Lutful Kabir et al., 2015; Sayyab et al., 2016), and these genes and mutations were only candidates for future validation and studies on the genetic basis of high reproduction of raccoon dogs.

### Genomic basis for the diverse immune system

The raccoon dog is a well-known reservoir host for many pathogens (Aoyagi et al., 2000; Guan et al., 2003; Kauhala and Kowalczyk, 2011; Kjaer et al., 2021; Qi et al., 2009; Song et al., 2019), but raccoon dogs with many pathogens are often asymptomatic, without signs of illness (Han et al., 2010; Yang et al., 2021b). We expected that the raccoon dog has a diverse immune system. We found several significantly enriched KEGG pathways related to the immune system. The base excision repair pathway is essential for the diversification of antigen receptors that shape the adaptive immune response (Stratigopoulou et al., 2020). Necroptosis can release danger signals to provoke the immune system for the clearance of pathogens, and can affect innate immunity by actively inducing the death of infected cells (Cho, 2020). The spliceosome is reported to have links with immune signaling, and some core spliceosome components present immune functions in cells (Yang et al., 2021a). The proteasome is considered necessary for the important immune functions of activated CD4^+^ T cells and proteasome inhibition suppresses unwanted and deregulated immune responses mediated by T cells (Berges et al., 2008). Although the primary functions of these enriched KEGG pathways are not immune functions, it is still possible to improve the immune system of raccoon dogs. Interestingly, a large proportion (14.56%) of PSGs were found to be immune-related, which was much large than the proportions for other functions. The functions of these genes are widely related to many innate and adaptive immune system processes (Table S19). Interestingly, raccoon dog-specific amino acid changes were found in 18 of the 30 immune genes and seemed to be retained by positive selection (Tables S20 and S21 and Figure S9). Further population genomic analysis showed that most of these amino acid changes were fixed in the population, even though the genetic diversity of the raccoon dog was high (Figure 5A), indicating the potential advantages of these mutations to raccoon dog survival. We did not predict the effects of these mutations on the protein function, because it is hard to conclude that a single mutation is harmful or helpful to the function of a certain protein. However, multiple mutations in a single gene may collaborate to aid their immune adaptation to new habitats, but this prediction should still be experimentally validated in future works. Although certain types of cancer have been reported in raccoon dogs, we did not find a comprehensive study on the investigation of the cancer incidence statistics in this species. A series of tumor suppressor genes found in the PSGs indicated the possible strong cancer survival ability of raccoon dogs.

It is energetically very demanding for the immune system to defend against pathogens. The synthesis of a cytokine requires the breakdown of 1,150 glucose molecules to generate 2,300 ATP molecules (Straub et al., 2010). The energy demand of the immune system usually represents 25%–30% of basal metabolism (Hortova-Kohoutkova et al., 2021; Straub et al., 2010). Balancing the energetic trade-off between immunity and other physiological energy-consuming processes is critically important for animal survival (Ganeshan et al., 2019). The cost of the immune response for innate and adaptive immunity is variable. The systemic innate immune response is the most resource-demanding, followed by cell-mediated immunity, and humoral responses cost the least when compared with innate and cell-mediated defenses due to inflammation being rarely induced (Lee and Klasing, 2004). A strategy to favor a less costly adaptive immune system and reduce the high resource-demanding innate immune response should be beneficial for allocating more energy for reproduction and growth (Lee and Klasing, 2004). However, we found that most PSGs (immune-related) were involved in innate immunity and induced an inflammatory response with fever, a loss of appetite and fatigue, which was energetically expensive. The large number of expanded gene families and PSGs related to energy metabolism in the raccoon dog genome may play important roles in supplying energy for their immune response.

### Several genetic factors may be jointly involved in the winter sleep of raccoon dogs

The most unique characteristic of raccoon dogs among canids is winter sleep (Asikainen et al., 2004). Raccoon dogs will fatten themselves before winter sleep (Nieminen et al., 2002). First, the omnivorous diet is very conducive to raccoon dogs to foraging and fattening. Except for taste receptor and detoxification-related genes, the two enriched olfactory transduction and salivary secretion pathways were also closely related to foraging behaviors and digestion (Jiahuan et al., 2018; Pedersen et al., 2018). The *GOLGB1* gene plays a crucial role in the development of the mammalian palate (Lan et al., 2016). The *HCRTR1* gene is the receptor gene of orexin, which is involved in foraging behavior and the intake of energy-dense food (Barson, 2020). The *MLN* gene encodes a small peptide hormone, *motilin*, which is secreted by the small intestine and regulates gastrointestinal motility and contractions, and stimulates hunger signaling (Tack et al., 2016). A genome-wide association study also detected a significant variant that was closest to the *MLN* gene in a study of the seasonal onset of hibernation in the 13-lined ground squirrel (Grabek et al., 2019). In addition, we also found that several lipid metabolism-related genes were under positive selection. The *SEC16B* gene in mice is required for lipid absorption, and is reported to be closely related to obesity in the human population (Sahibdeen et al., 2018; Shi et al., 2021). The *BSCL2* gene is also involved in lipid metabolism, and the deletion of *BSCL2* in mature white and brown adipose tissue triggers cAMP/PKA-mediated lipolysis and fatty acid oxidation resulting in adipose tissue loss (Zhou et al., 2020, 2022). These genes may facilitate intensive foraging and fat accumulation before winter sleep.

Unlike typical hibernation, the body temperature of raccoon dogs is close to normal during winter sleep (Asikainen et al., 2004). We found that the expanded gene families were enriched in thermogenesis (map04714, p = 8.36E-10) which may help raccoon dogs to maintain a near-normal body temperature. During winter sleep, fuel use shifts from glucose to lipids. Bears exhibit insulin resistance during hibernation and regain insulin sensitivity in the spring (Rigano et al., 2017). The positively selected gene *STXBP5L* is reported to be a negative regulator of insulin secretion, which may be involved in the usage shift of fuel in raccoon dogs during winter sleep. In addition, a large number of PSGs related to immunity may play an important role in preventing raccoon dog from becoming infected with the pathogen during winter sleep.

### Historical declining population and currently high genetic diversity

Although the raccoon dog is considered a successful invasive species with a strong ability to survive, its effective population size was detected to be continuously declining from ∼50 kya. The population decline accelerated from ∼28 kya to 11 kya, which may be caused by the harsh climate during the Last Glacial Maximum (Denton et al., 2010). However, despite the climate becoming warmer at the beginning of the Holocene, the effective size of the raccoon dog population was still declining. Considering the frequent human activity within the most recent 10 kya, we speculate that the population decline of the raccoon dog in its evolutionary history might in part be due to human activities and climate change. In addition, changes in predator and prey abundance, habitat connectivity, and habitat productivity may also influence the fluctuation of the raccoon dog population.

Generally, population size is positively correlated with the level of genetic diversity (Ouborg et al., 2010). Interestingly, the genetic diversity of the raccoon dog is high, which was consistent with previous reports (Lavrov, 1971; Pitra et al., 2009), indicating a current healthy population. The historical long-term population decline of this species seems to indicate that this species may not be as tolerant and plastic as we expected for such a successful invader. The success of invasion of a certain species is not directly related to its population size but is closely related to the “preadapted” characteristics and post-invasive adaptation (North et al., 2021). We cannot investigate the post-invasive adaptation of raccoon dogs after invasion due to the lack of global samples. The “preadapted” biological characteristics, such as an omnivorous diet, diverse immune system, high reproduction rate, and winter sleep, could contribute to their successful invasiveness, even though raccoon dogs are experiencing a long-term population decline.

### Limitations of the study

Although we assembled a high-quality chromosome-scale genome of the raccoon dog, we did not assemble the B chromosomes, which may contribute to important biological functions. In addition, functional analysis should be further performed to validate the candidate genes and raccoon dog-specific mutations we found in this study to be associated with omnivory, reproduction, and immunity.

## STAR★Methods

### Key resources table

**Table:** 

REAGENT or RESOURCE	SOURCE	IDENTIFIER
**Chemicals, peptides, and recombinant proteins**		

TRlzol reagent	Invitrogen, USA	Cat#15596 - 026

**Critical commercial assays**		

Monarch HMW DNA Extraction Kit	NEB, Ipswich, England	Cat#T3060L
SMRTbell Template Prep Kit 1.0	Pacific Biosciences, CA, USA	Cat#100-259-100
Qiagen Blood & Cell Culture DNA Mini Kit	Qiagen, United States	Cat#13323

**Deposited data**		

*Nyctereutes procyonoides* reference genome	This paper	CNSA: CNP0002053
*Bos Taurus* reference genome	Ensembl	ARS-UCD1.2
*Ursus thibetanus thibetanus* reference genome	Ensembl	ASM966005v1
*Acinonyx jubatus* reference genome	NCBI	GCF_003709585.1_Aci_jub_2
*Ursus arctos* reference genome	NCBI	GCF_003584765.1_ASM358476v1
*Sus scrofa* reference genome	Ensembl	Sscrofa11.1
*Panthera leo* reference genome	Ensembl	PanLeo1.0
*Panthera pardus* reference genome	Ensembl	PanPar1.0
*Vulpes lagopus* reference genome	NCBI	GCF_018345385.1_ASM1834538v1
*Ursus maritimus* reference genome	Ensembl	UrsMar_1.0
*Canis lupus dingo* reference genome	Ensembl	ASM325472v1
*Panthera tigris altaica* reference genome	Ensembl	PanTig1.0
*Vulpes Vulpes* reference genome	Ensembl	VulVul2.2
*Homo sapiens* reference genome	Ensembl	GRCh38
*Felis catus* reference genome	Ensembl	Felis_catus_9.0
*Equus caballus* reference genome	Ensembl	EquCab3.0
*Canis lupus familiaris* reference genome	Ensembl	CanFam3.1
*Oryctolagus cuniculus* reference genome	Ensembl	OryCun2.0

**Software and algorithms**		

Canu (v2.0)	(Koren et al., 2017)	https://github.com/marbl/canu
NextPolish (v1.3.1)	(Hu et al., 2020)	https://github.com/Nextomics/NextPolish
Purge_dups (v1.2.5)	(Guan et al., 2020)	https://github.com/dfguan/purge_dups
LRScaf (v1.1.8)	(Qin et al., 2019)	https://github.com/shingocat/lrscaf
BWA (v0.7.17)	(Li and Durbin, 2010)	http://bio-bwa.sourceforge.net/
Juicer (v1.5)	(Durand et al., 2016)	https://github.com/aidenlab/juicer
3d-DNA (v190716)	(Dudchenko et al., 2017)	https://github.com/aidenlab/3d-dna
BUSCO (v5.2.2)	(Manni et al., 2021)	https://busco.ezlab.org/
LTR finder (v1.0.6)	(Xu and Wang, 2007)	https://github.com/xzhub/LTR_Finder
MITE-hunter (v4.07)	(Han and Wessler, 2010)	https://github.com/jburnette/MITE-Hunter
RepeatModeler2 (v2.0.1)	(Flynn et al., 2020)	http://www.repeatmasker.org/RepeatModeler/
RepeatMasker (v4.0.5)	(Tarailo-Graovac and Chen, 2009)	https://www.repeatmasker.org/
Tandem Repeats Finder (v4.07)	(Benson, 1999)	https://tandem.bu.edu/trf/trf.html
GlimmerHMM (v3.0.1)	(Majoros et al., 2004)	https://ccb.jhu.edu/software/glimmerhmm/
Augustus (v3.0.3)	(Stanke et al., 2004)	https://bioinf.uni-greifswald.de/augustus/
SNAP (v11/29/2013)	(Korf, 2004)	https://github.com/KorfLab/SNAP
Trimmomatic (v0.30)	(Bolger et al., 2014)	http://www.usadellab.org/cms/?page=trimmomatic
Trinity (v2.13.2)	(Haas et al., 2013)	https://github.com/trinityrnaseq/trinityrnaseq
PASA (v2.0.2)	(Haas et al., 2008)	https://github.com/PASApipeline/PASApipeline
Blastall (v2.2.26)	(Mount, 2007)	http://gensoft.pasteur.fr/docs/blast/2.2.26/
GeneWise (v2.4.1)	(Birney et al., 2004)	https://www.ebi.ac.uk/Tools/psa/genewise/
MAKER pipeline (v3.01.03)	(Campbell et al., 2014)	https://www.yandell-lab.org/software/maker.html
tRNAscan-SE (v2.0.9)	(Lowe and Eddy, 1997)	https://github.com/UCSC-LoweLab/tRNAscan-SE
INFERNAL (v1.1.1)	(Nawrocki et al., 2009)	http://eddylab.org/infernal/
InterProScan (v5.52-86.0)	(Jones et al., 2014)	https://github.com/ebi-pf-team/interproscan
MAFFT (v.7.310)	(Katoh and Standley, 2013)	https://mafft.cbrc.jp/alignment/software/
PAL2NAL (v14)	(Suyama et al., 2006)	http://www.bork.embl.de/pal2nal/
Trimal (v1.4.1)	(Capella-Gutierrez et al., 2009)	http://trimal.cgenomics.org/
IQTREE (v1.6.12)	(Nguyen et al., 2015)	http://www.iqtree.org/
Treefam (v1.4)	(Li et al., 2006)	http://www.treefam.org/
CAFE (v4.2.1)	(De Bie et al., 2006)	https://hahnlab.github.io/CAFE/src_docs/html/index.html
PAML (v4.8)	(Yang, 2007)	http://evomics.org/resources/software/molecular-evolution-software/paml/
Picard (v2.1.1)	N/A	https://github.com/broadinstitute/picard
Sentieon (v202010.01)	(Freed et al., 2017)	https://support.sentieon.com
VCFtools (v4.1)	(Danecek et al., 2011)	http://vcftools.sourceforge.net/
PSMC (v0.6.5)	(Li and Durbin, 2011)	https://github.com/lh3/psmc
MSMC2 (v2.1.1)	(Schiffels and Durbin, 2014)	https://github.com/stschiff/msmc2
SMC++ (v1.15.4)	(Terhorst et al., 2017)	https://github.com/popgenmethods/smcpp

**Other**		

Raw and analyzed data	This paper	CNSA: CNP0002053

### Resource availability

#### Lead contact

Further information and requests for reagents should be directed to and will be fulfilled by the Lead Contact, Prof. Zhijun Hou (houzhijundb@163.com).

#### Materials availability

This study did not generate new unique reagents.

### Experimental model and subject details

An adult male raccoon dog (one-year-old) who died of natural causes was collected from Harbin, Heilongjiang, China for the genome assembly. This individual is captive born and from the subspecies of *N. procyonoides ussuriensis*. We immediately stored tissue samples in liquid nitrogen after quick dissection to perform high-quality DNA and RNA isolation. Samples from 5 organs, including the heart, lung, spleen, liver, and kidney were used for RNA sequencing. The muscle sample was used for Pacific Bioscience (PacBio) sequencing and genome survey. The liver sample was used for Hi-C sequencing. We also collected blood samples of another 38 individuals from Harbin Hualong Fur Farm for whole genome resequencing. Sample collection, experiments and research design in this study were all approved by the Institutional Review Board of BGI (BGI-IRB E22001). We strictly followed the guidelines from BGI-IRB for all procedures conducted in this study.

### Method details

#### Nucleic acid extraction, DNA library preparation and sequencing

High-molecular-weight genomic DNA was isolated from the muscle sample using the Monarch HMW DNA Extraction Kit (NEB, Ipswich, England). The SMRTbell Template Prep Kit 1.0 (Pacific Biosciences, CA, USA) was used for SMRTbell library preparation based on DNA fragments with an average size of 20 kb. To obtain the 20 kb DNA fragments, we first extracted the total genomic DNA and detected the completeness of the DNA by agarose gel electrophoresis, and then the genomic DNA was randomly broken into fragments of about 20 kp length by Covaris ultrasonic crusher. The DNA library was then subjected to the PacBio Sequel II platform for long-read sequencing. For Hi-C library construction, we first performed the crosslinking with formaldehyde for the liver sample, and then one Hi-C library was constructed by using the dpnII restriction endonuclease. Genomic DNAs from the 38 blood samples for resequencing and the muscle sample for genome survey were isolated with Qiagen Blood & Cell Culture DNA Mini Kit (Qiagen, United States). The 5 organs were used for RNA isolated with TRlzol reagent (Invitrogen, USA) following the manufacturer’s guidelines. The extracted RNA was then fragmented into 200–400 bp by heat digestion with a divalent metal cation by adjusting the time of incubation, and then reverse-transcribed to cDNA for library preparation. 45 short insert size libraries (5 for cDNA, 40 for genomic DNA) were finally constructed according to the manufacturer’s instruction of the MGI platform (MGI, Shenzhen, China). All 45 libraries were finally sequenced on the DNBSEQ-T1 platform with 100-bp paired-end sequencing strategy.

#### Genome assembly, annotation, and assessment

To estimate the genome size of the raccoon dog, we calculated the distribution of the frequency of single nucleotide depth across the genome with 82.21 Gb short reads. The genome size was estimated by dividing the peak depth by the total sequencing base number (Pflug et al., 2020). The primary genome was assembled by Canu (Koren et al., 2017) (v2.0) with PacBio long reads. This primary assembly was polished with both DNBSEQ short reads and PacBio long reads by three rounds using NextPolish (Hu et al., 2020) (v1.3.1) to correct sequence errors. We used purge_dups (Guan et al., 2020) (v1.2.5) to remove haplotypic duplication through sequence similarity and read depth. LRScaf (Qin et al., 2019) (v1.1.8) was then used for the linking of contigs into longer scaffolds. To obtain the chromosome-scale genome, we mapped Hi-C reads to the primary genome by Burrows-Wheeler Aligner (BWA, v0.7.17) (Li and Durbin, 2010), and then used Juicer (Durand et al., 2016) (v1.5) for Hi-C data quality control, finally used 3d-DNA pipeline (Dudchenko et al., 2017) (v190716) to concatenate the scaffolds to the chromosome-scale genome. BUSCO (Manni et al., 2021) (v5.2.2) analysis was performed to evaluate the completeness of the final chromosome-scale genome with both laurasiatheria_odb10 and mammalia_odb10 data set.

A combination of *de novo* and homolog-based methods was used for repeat annotation. We firstly used the LTR finder (Xu and Wang, 2007) (v1.0.6), MITE-hunter (Han and Wessler, 2010) (v4.07), and RepeatModeler2 (Flynn et al., 2020) (v2.0.1) to annotate *de novo* repeats. These identified repeats were then merged into the RepBase as known elements. Finally, we performed RepeatMasker (Tarailo-Graovac and Chen, 2009) (v4.0.5) to classify and identify transposable elements with a conserved BLASTN search against the prepared RepBase library. Tandem repeats were also annotated by Tandem Repeats Finder (Benson, 1999) (v4.07) software. We then masked all repeat elements for gene annotation.

Protein-coding genes were predicted using *de novo*, homology-based and transcript mapping approaches. GlimmerHMM (Majoros et al., 2004) (v3.0.1), Augustus (Stanke et al., 2004) (v3.0.3), and SNAP (Korf, 2004) (v11/29/2013) were used for *de novo* gene prediction. For RNA-seq based prediction, we first filtered RNA-seq data by Trimmomatic (Bolger et al., 2014) (v0.30). Transcripts were assembled using Trinity (Haas et al., 2013) (v2.13.2) based on clean RNA-seq data. Program to Assemble Spliced Alignments (PASA) (Haas et al., 2008) (v2.0.2) was finally used to align transcript against the raccoon dog genome to obtain gene structures. Homology-based prediction was carried out by mapping protein sequences of UniProt database (release-2020_05), *Homo sapiens*, *Mus musculus*, *Canis lupus familiaris*, *Nyctereutes procyonoides* (Chueca et al., 2021), and *Vulpes vulpes* to the raccoon dog genome using the Blastall (Mount, 2007) (v2.2.26) with an E-value cut-off of 1e-5. Then the GeneWise (Birney et al., 2004) (v2.4.1) was used to predict gene models by analyzing alignment results. The final gene set representing RNA-seq, homology, and *de novo* predicted genes was generated by performing MAKER pipeline (Campbell et al., 2014) (v3.01.03). Blastall (Mount, 2007) (v2.2.26) was used to identify ribosomal RNA (rRNA) sequences by aligning human rRNA sequences to the raccoon dog genome. Transfer RNA (tRNA) was predicted by tRNAscan-SE (Lowe and Eddy, 1997) (v2.0.9). Both microRNA (miRNA) and small nuclear RNA (snRAN) were predicted using INFERNAL v1.1.1 (Nawrocki et al., 2009) by aligning the raccoon dog genome sequences to the Rfam database. Functional annotation was performed by performing BLAST search against the SwissProt, TrEMBL and Kyoto Encyclopedia of Genes and Genomes (KEGG) database with an E-value cut-off of 1e-5. InterProScan (Jones et al., 2014) (v5.52-86.0) was used to predict motifs and domains, as well as Gene ontology (GO) terms.

#### Phylogeny reconstruction with genome-wide single-copy genes

Homologous genes of 18 species were determined by performing all-to-all BLASTP analysis for proteins from each species with the parameter of “-evalue 1e-5”. We identified 6,936 single-copy genes shared by these 18 species. A phylogenetic tree was then constructed based on these single copy genes with the following procedures: 1) We used MAFFT (Katoh and Standley, 2013) (v.7.310) to do multiple amino acid sequence alignments for each single-copy gene orthogroup; 2) The aligned sequences of the amino acid were converted to aligned DNA sequences using PAL2NAL (Suyama et al., 2006) (v14); 3) Gaps were removed using the trimal (Capella-Gutierrez et al., 2009) (v1.4.1) software; 4) We calculated the best-fit substitution model using ModelFinder (Kalyaanamoorthy et al., 2017); 5) We constructed a maximum-likelihood (Warren et al.) phylogenetic tree by IQTREE (Nguyen et al., 2015) (v1.6.12) with concatenated super-genes. For the GST gene family, we also used the same methods to generate the ML phylogenetic tree with only Canidae species.

#### Gene family expansion and contraction

We clustered all annotated genes to identify gene families with hierarchically clustering on a sparse graph by the software Treefam (Li et al., 2006) (v1.4). We identified 20,699 gene families in the above-mentioned 18 genomes, and 16,403 were in the raccoon dog genome. We then used CAFE (De Bie et al., 2006) (v4.2.1) to detect contracted and expanded gene families. The random birth and death model was used to estimate the size of gene families at ancestral nodes. A family-wise p < 0.05 was set as the cut-off value. We carried out the KEGG and GO enrichment analysis on expanded gene families with all annotated genes as the background. To avoid an inaccurate chi-square test, we performed Fisher’s exact test with an expected gene count of below five. We finally adjusted p values using the Benjamini-Hochberg method (Benjamini and Yekutieli, 2005) with multiple tests by specifying a false discovery rate with q-value less than 0.05.

#### Positively selected genes (PSGs)

The phylogenetic tree and 6,936 single-copy genes identified in the section of “phylogeny reconstruction with genome-wide single-copy genes” were subjected to the selection analysis. PSGs were identified in the raccoon dog genome by comparing these single copy orthologs with the above-mentioned 17 species. We calculated the non-synonymous to synonymous substitutions ratio (dN/dS) under the branch-site model in the CodeML of PAML (Yang, 2007) (v4.8) software, with raccoon dog as the foreground. We performed the likelihood ratio test (LRT) for Lnl values of each model pair and obtained p values with chi-square test. The positively selected sites were identified with a false discovery rate corrected p value less than 0.05.

#### Genome-wide variants calling and quality control

We mapped the whole genome resequencing data of 38 individuals to the raccoon dog genome by using the BWA (Li, 2013) *mem* method with default parameters. We then used the Picard package (v2.1.1) for sorting, reordering and deduplication of alignment files for variants calling. Sentieon (Freed et al., 2017) (v202010.01) DNAseq Haplotyper was used to call variants for each sample independently to generate the genomic Variant Call Format (gVCF) files. The following joint variant calling was carried out by Sentieon DNAseq GVCFtyper with the 38 gVCF files to finally create a common VCF file. To facilitate downstream analysis, we removed InDels and multi-allelic variants and then performed hard filtering with “QD < 2.0 || FS > 60.0 || MQ < 40.0 || MQRankSum < −12.5 || ReadPosRankSum < −8.0 --filter-name snp_filter”(DePristo et al., 2011).

#### Genomic diversity and population demography

Autosomal variants were selected to calculate the genome-wide *H* and genetic diversity (π) using VCFtools (Danecek et al., 2011) (v4.1). The population level π was calculated with 500-kb sliding window by setting the parameter “*-window-pi 500000*”. For inference of population history, we first used the Pairwise Sequentially Markovian Coalescent (PSMC) (Li and Durbin, 2011) (v0.6.5) to infer the fluctuation of effective population size over its evolutionary history under the pattern of “4 + 25×2 + 4+6”. To show the robustness of this estimation, we performed 100 bootstrap replicates for the same individual. We visualized the PSMC result by scaling the time to the real years by using the mutation rate (μ) of 1.0 × 10^−8^ substitution per site per generation and a generation time (g) of three years (Freedman et al., 2014). PSMC has been shown to be inappropriate for inferring more recent population history (e.g. more recent than 20 kya for human populations) (Li and Durbin, 2011), Therefore, we performed MSMC2 (Schiffels and Durbin, 2014) (v2.1.1) to infer the more recent population history (especially the time period from 1 kya to 10 kya) of the raccoon dog with four randomly selected individuals. Considering switch errors induced by the phasing process which will further bias the MSMC2 inference, we also performed the SMC++ (Terhorst et al., 2017) (v1.15.4) analysis based on the population variations from all 38 individuals to validate the result from MSMC2. For both MSMC2 and SMC++, we used the same generation interval and mutation rate used in the PSMC to show the results.

### Quantification and statistical analysis

Quantification and statistical analysis used in the genome assembly and comparative genome analysis can be found in the method details.

## Data Availability

The data that support the findings in this study have been deposited into CNGB Sequence Archive (CNSA) (Guo et al., 2020) of China National GeneBank DataBase (CNGBdb) (Chen et al., 2020) with accession number CNP0002053. All original codes are included in the supplemental information. Any additional information required to reanalyze the data reported in this paper is available from the lead contact upon request.

## Supporting citations

The following reference appears in the supplemental information: Abascal et al., 2016; Anthoni et al., 2007; Arroyo et al., 2016; Aslanukov et al., 2005; Bhatnagar et al., 2011; CABI, 2019; Cho et al., 2013; Coppo et al., 2016; Corbett-Detig et al., 2015; Cotton et al., 2022; Demeyer et al., 2019; Ding et al., 2020; Dobrynin et al., 2015; Dolgachev et al., 2018; Funakoshi et al., 2015; Guo et al., 2021; Gutekunst et al., 2018; Hitomi et al., 2019; Huber et al., 2008; Ikutani et al., 2012; Jacquet et al., 2020; Jamsai et al., 2017; Kallianpur et al., 2014; Keckesova et al., 2017; Kisiel et al., 2013; Korman et al., 2008; Lee et al., 2019; Leffler et al., 2012; Li et al., 2021; Lindblad-Toh et al., 2005; Liu et al., 2015; Maciejewska-Skrendo et al., 2019; Mehawej et al., 2020; Mikkelsen et al., 2007; Moore et al., 2008; Murphy, 2020; Nishimura et al., 2021; Nishioka et al., 2002; Palazon et al., 2014; Palmer et al., 2017; Peris-Moreno et al., 2020; Pickering and Booty, 2021; Qin et al., 2020; Ranasinghe and Eri, 2018; Readinger et al., 2009; Robinson et al., 2016; Ryan et al., 2012; Salzer et al., 2016; Santoni et al., 2013; Schenkel et al., 2007; Seo et al., 2005; Sheikh et al., 2015; Shema et al., 2008; Silver and Livingston, 2012; Smith, 2010; Strobl et al., 2011; Tsuchiya et al., 2021; Tuo et al., 2019; Valizadeh et al., 2015; Wang et al., 2016; Wang et al., 2018; Warren et al., 2015; Yang et al., 2016; Yoshinaka et al., 2019; Zhan et al., 2020; Zhang et al., 2012; Zhang et al., 2013; Zhao et al., 2017; Zhu et al., 2018.

## Supplemental information

Document S1. Figures S1–S10, Tables S1–S8, S11–S13, S15, S16, S19–S24, and S26–S29

Table S9. Sequencing depths of all scaffolds of the Raccoon dog assembly, related to Figure 2

Table S10. The heterozygosity (H) of the 26 autosomes, X chromosome and Y-linked scaffold of 38 requencing individuals, related to Figure 2

Table S14. The GO enrichment result of expanded families in the raccoon dog genome compared with 17 species, related to Figure 3

Table S17. The positively selected gene list calculated by PAML with other 17 species, related to STAR Methods

Table S18. The prediction of functional changes for genes with raccoon dog specific amino acid changes by using the PROVEAN method, related to Figure 4

Table S25. The positively selected gene list calculated by PAML with other 14 non-hibernating species, related to STAR Methods
