# Supplementary material for: The chromosome-scale genome of the raccoon dog: Insights into its evolutionary characteristics
Source: iScience. 2022 Sep 15;25(10):105117. doi: 10.1016/j.isci.2022.105117 (PMC9523411; doi:10.1016/j.isci.2022.105117)
Supplement: Document S1. Figures S1–S10, Tables S1–S8, S11–S13, S15, S16, S19–S24, and S26–S29 [file mmc1.pdf]

iScience, Volume *25*

Supplemental information

The chromosome-scale genome of the raccoon dog: Insights into its evolutionary characteristics

Tianming Lan, Haimeng Li, Shangchen Yang, Minhui Shi, Lei Han, Sunil Kumar Sahu, Yaxian Lu, Jiangang Wang, Mengchao Zhou, Hui Liu, Junxuan Huang, Qing Wang, Yixin Zhu, Li Wang, Yanchun Xu, Chuyu Lin, Huan Liu, and Zhijun Hou

Supplementary figure 1. Contig contact matrix of the raccoon dog genome. The Hi-C interaction density is represented by the color depth, related to the STAR Methods.

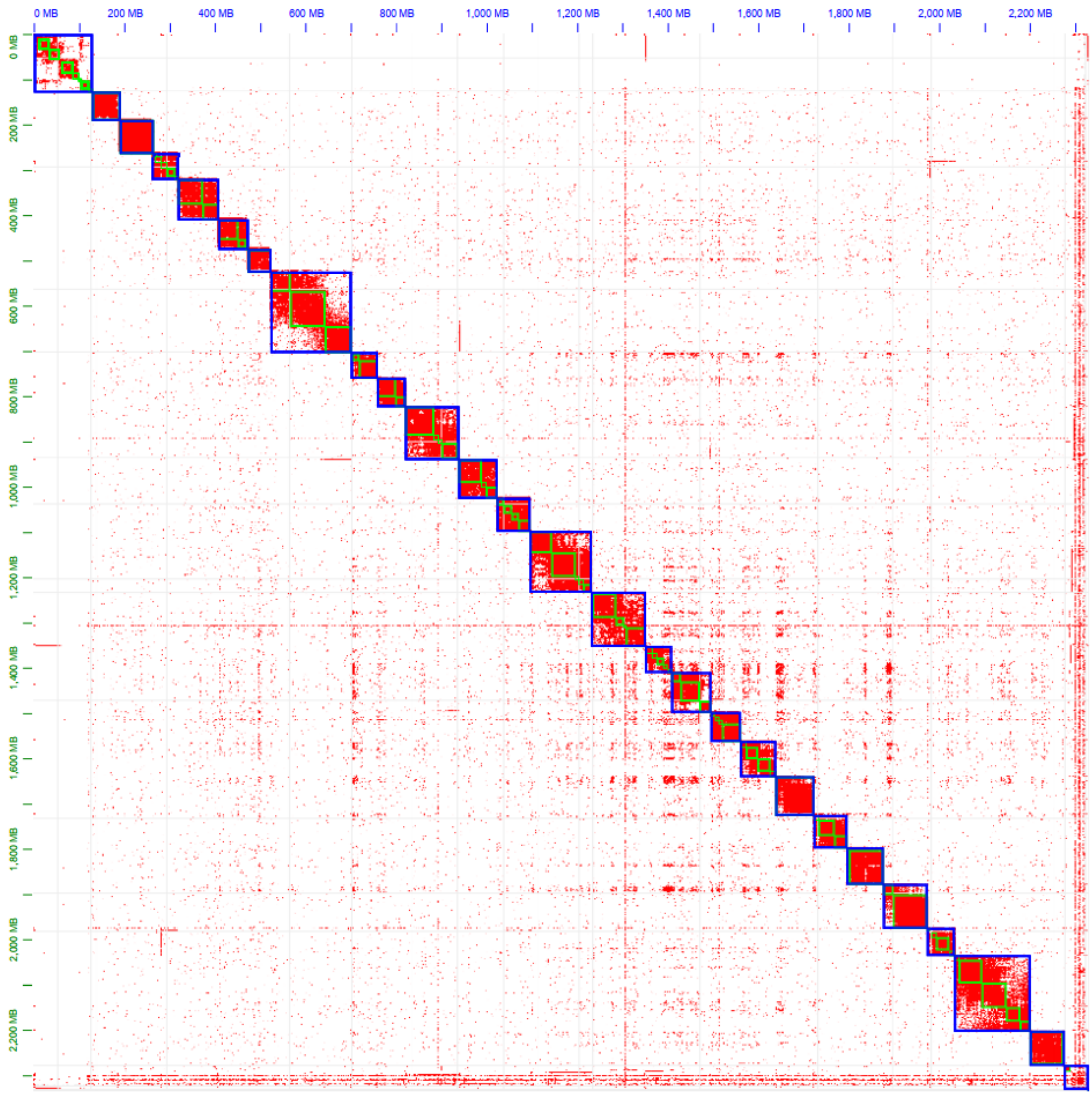

Supplementary figure 2. Population-scale π-values across 27 chromosomes in the raccoon dog genome, related to Figure 1B.

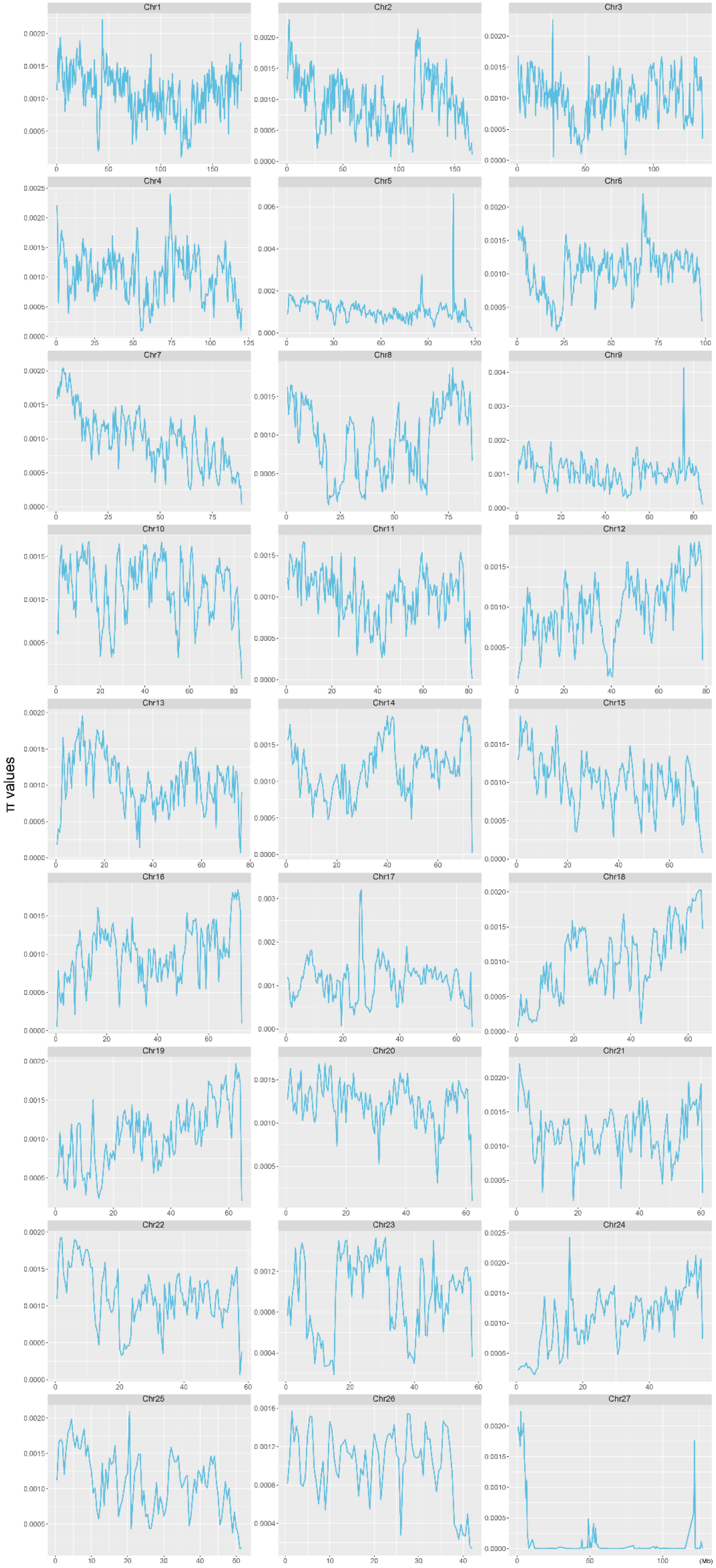

Supplementary figure 3. SNP counts calculated by 500 kb window across 27 chromosomes in the raccoon dog genome, related to Figure 1B.

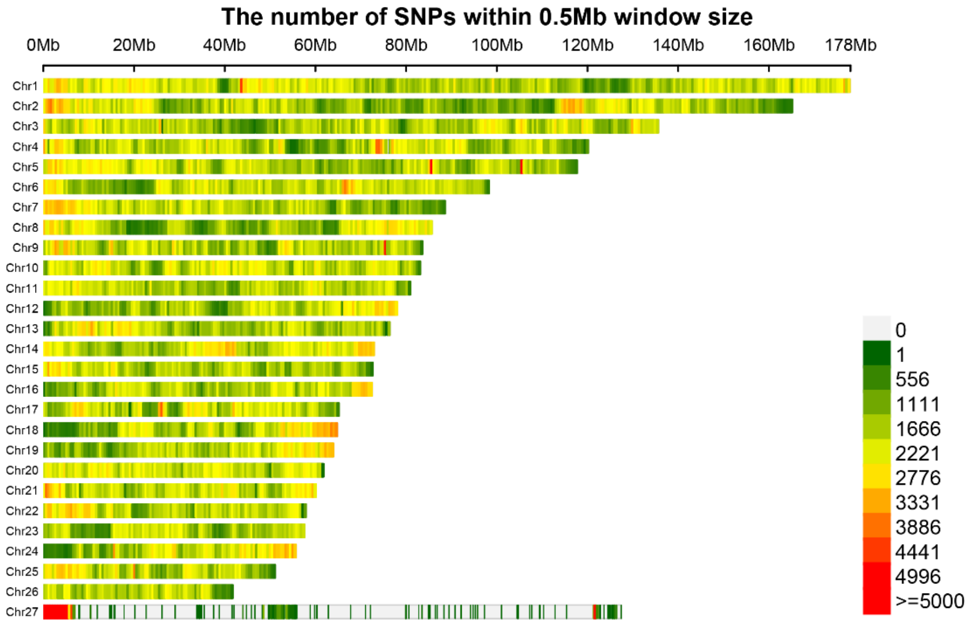

Supplementary figure 4. Sequencing depth (X) and GC content (%) calculated by 500 kb window across 27 chromosomes in the racoon dog genome, related to Figure 1B. The blue line indicates the sequencing depth (X) and the red line indicates the GC content (%).

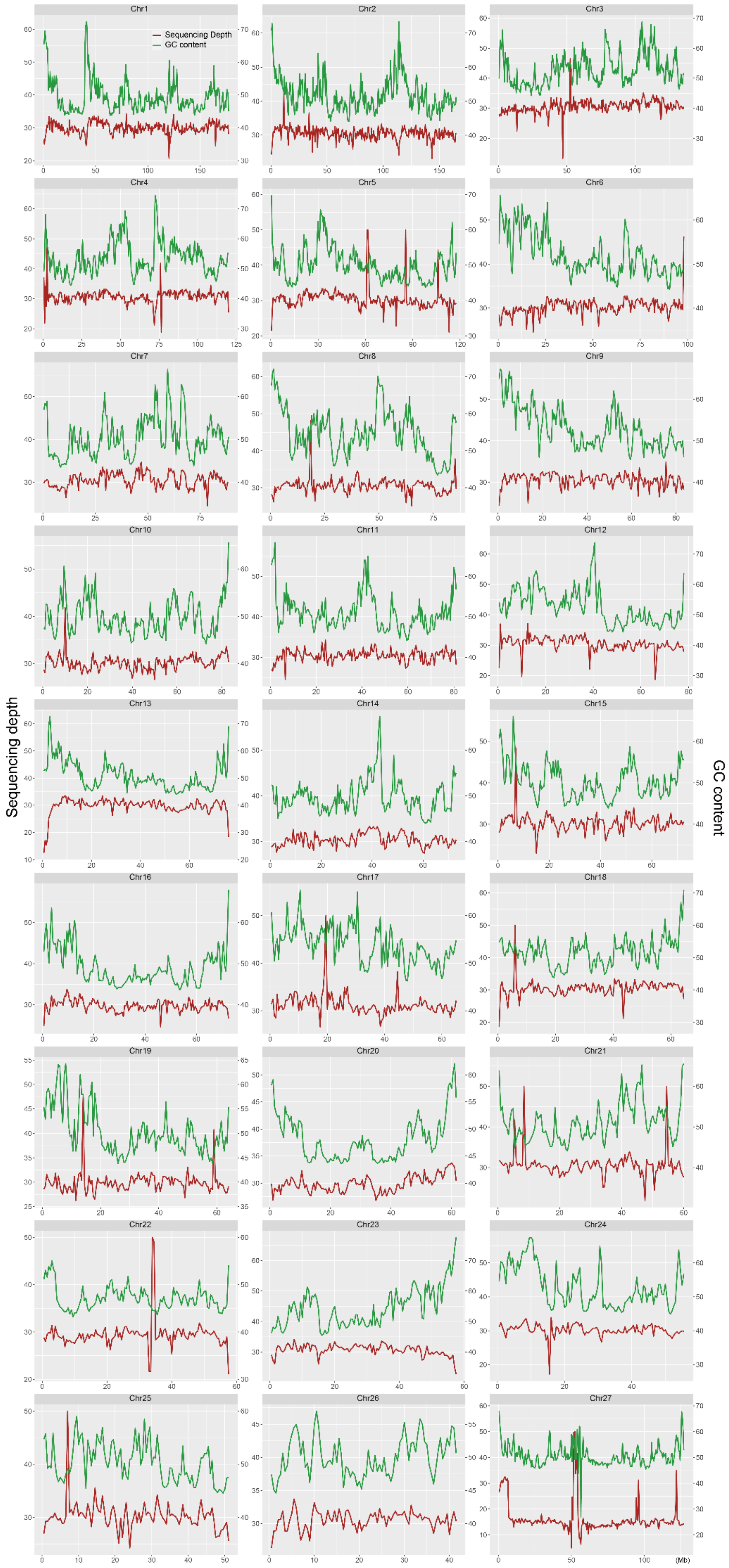

Supplementary figure 5. Gene density calculated by 500kb window for the 27 chromosomes in the raccoon dog genome, related to Figure 1B.

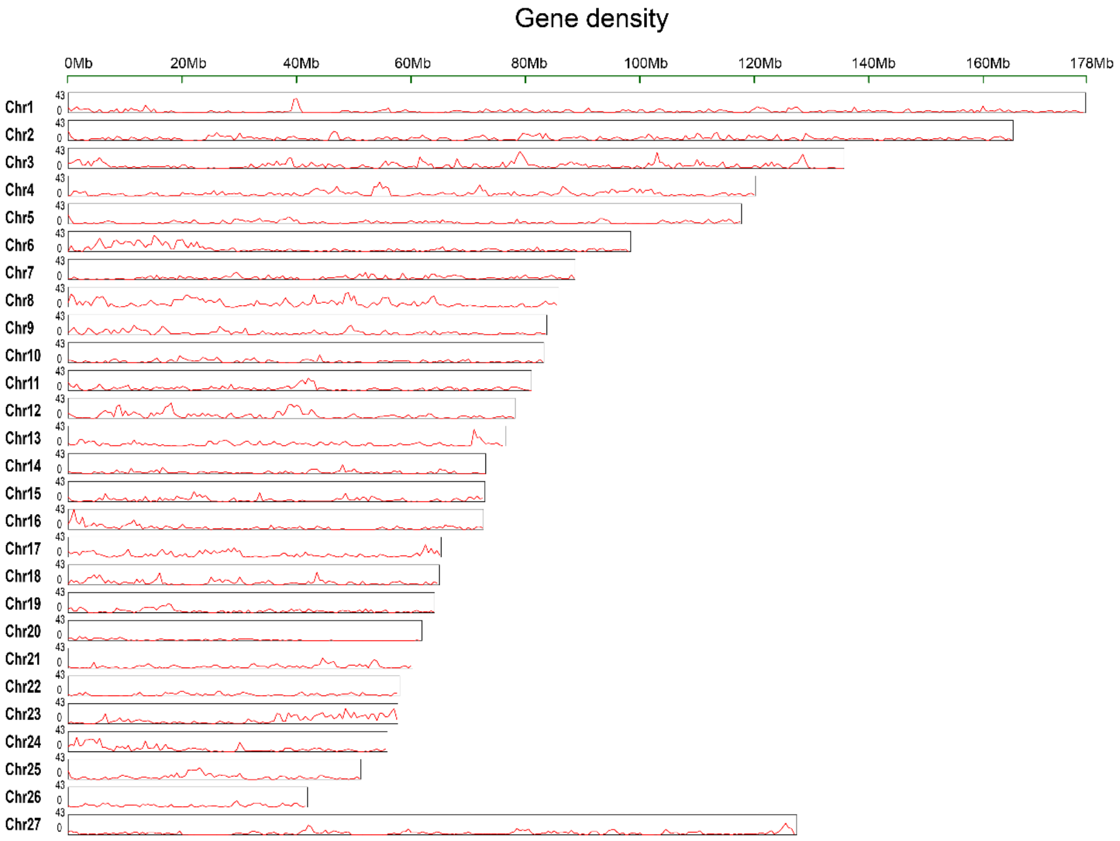

Supplementary figure 6. Comparisons of CDS length, mRNA length, intron length and exon length among 6 species, related to the STAR Methods. Hsap: human; Mmus: mouse; Clup: dog; Lcan: lynx; Fcat: cat; Npro: raccoon dog.

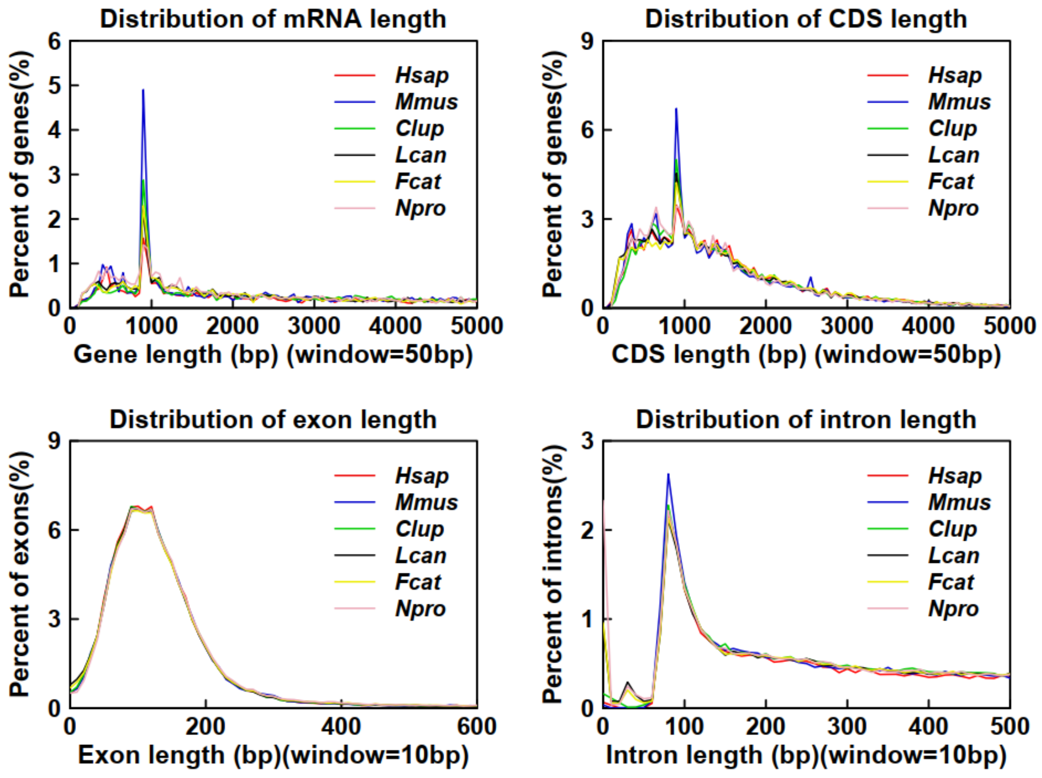

Supplementary figure 7. Venn diagram representing the functional annotation of the raccoon dog gene set, related to the STAR Methods.

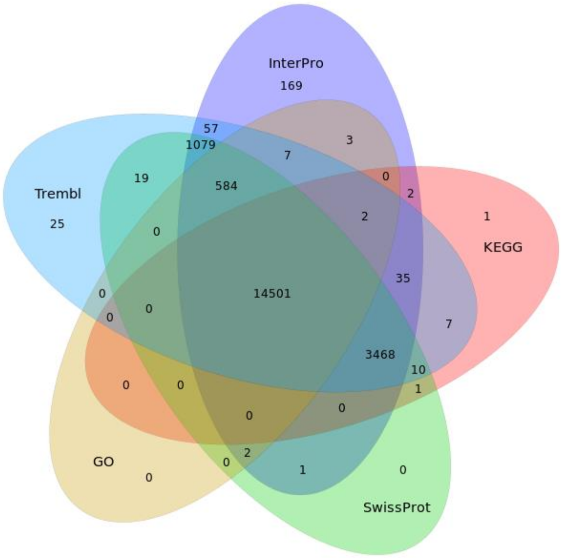

Supplementary figure 8. Sequencing depths of the 26 autosomes, X chromosome (Chr27) and the Y-linked scaffold (Scaffold30), related to Figure 2.

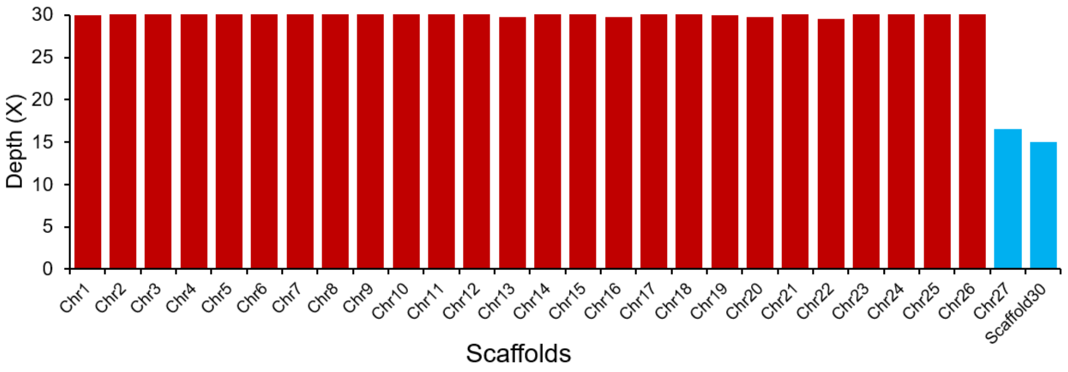

Supplementary figure 9. Raccoon dog specific amino acid changes compared with human and other canids on 18 immune related genes, related to the STAR Methods.

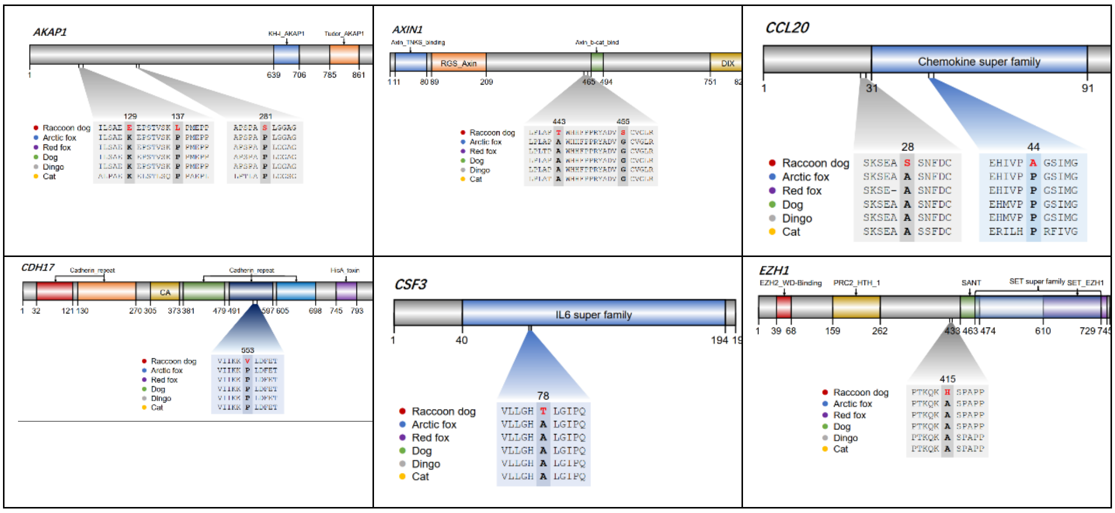

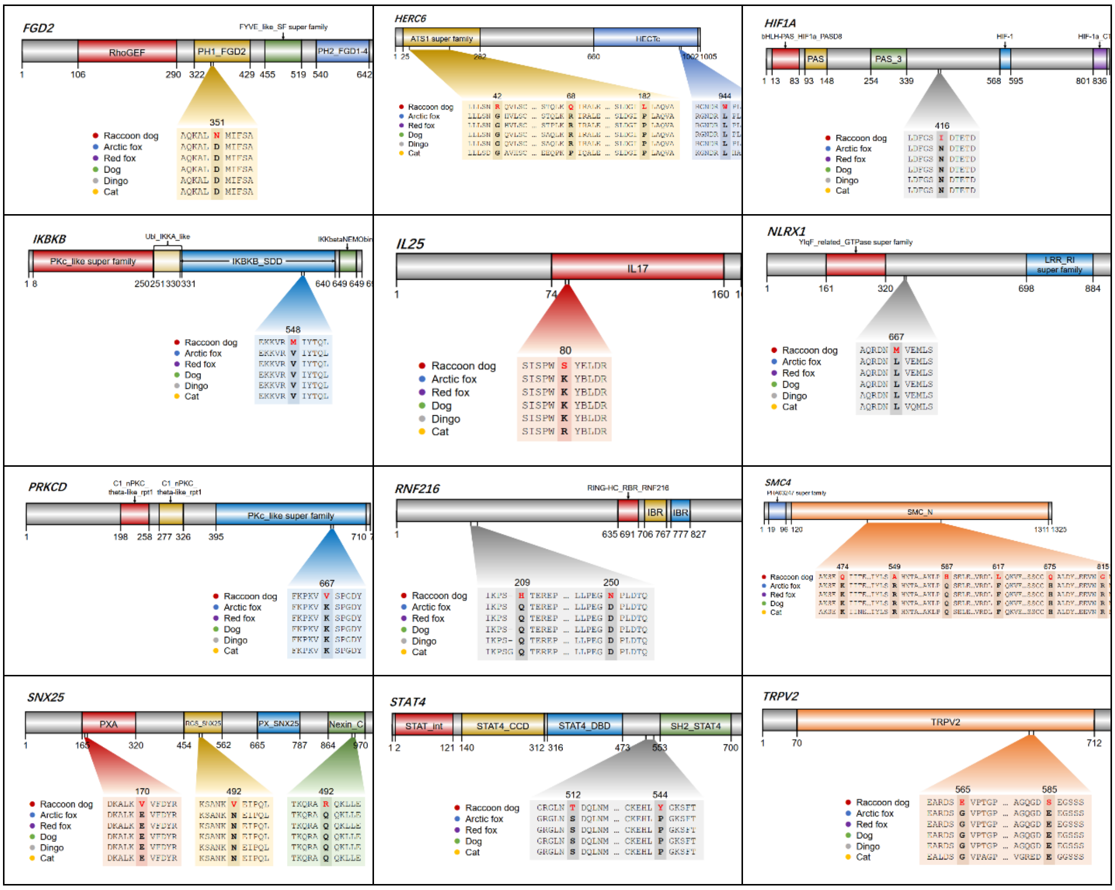

Supplementary figure 10. Genome size estimation by the distribution of the frequency of single nucleotide depth across the genome, related to the STAR Methods.

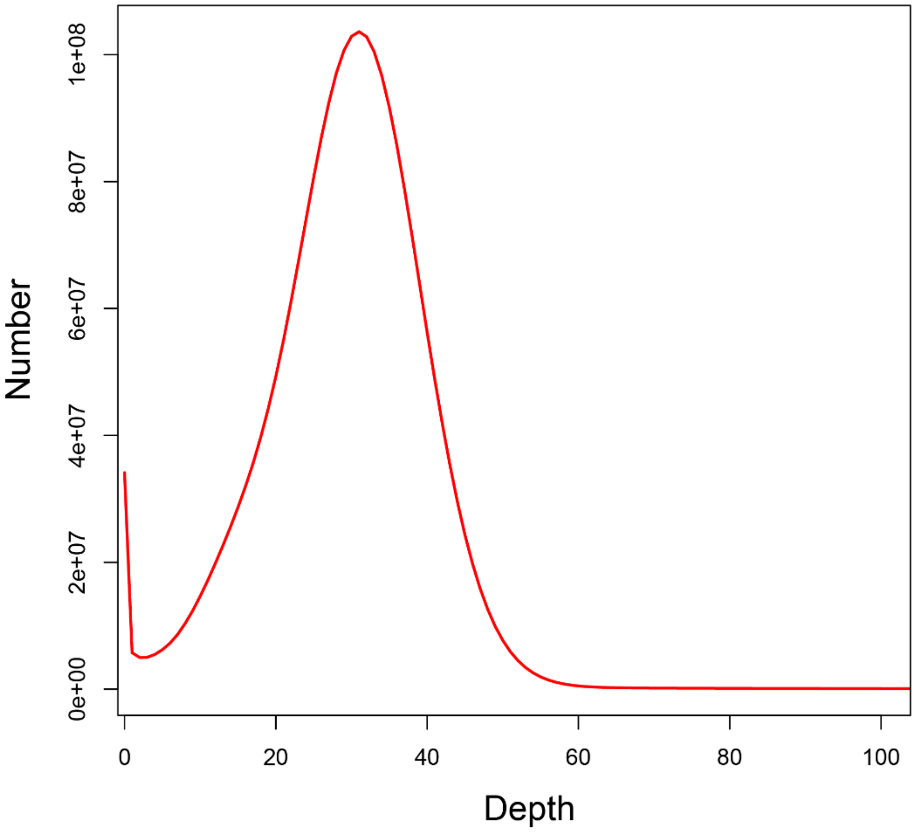

Supplementary table 1. The length of 27 chromosomes assembled for the raccoon dog, related to Figure 1B.

Chromosome	Length (bp)
Chr1	177,962,720
Chr2	165,264,888
Chr3	135,712,509
Chr4	120,253,313
Chr5	117,787,393
Chr6	98,395,122
Chr7	88,677,575
Chr8	85,782,641
Chr9	83,697,973
Chr10	83,210,897
Chr11	81,017,826
Chr12	78,174,188
Chr13	76,488,698
Chr14	73,023,569
Chr15	72,894,907
Chr16	72,619,702
Chr17	65,246,759
Chr18	64,938,764
Chr19	64,068,175
Chr20	61,885,687
Chr21	60,260,761
Chr22	57,993,334
Chr23	57,659,079
Chr24	55,826,000
Chr25	51,198,383
Chr26	41,869,042
Chr27	127,439,728

Supplementary table 2. Result of BUSCO analysis of the Raccoon dog genome, related to the STAR Methods.

**Level**	**BUSCO assessment results**	**Database**	**Parameter**
Genome	C:96.2%[S:93.5%,D:2.7%],F:0.9%,M:2.9%,n:12234	laurasiatheria_odb10	-m genome
Genome	C:96.4%[S:94.2%,D:2.2%],F:0.8%,M:2.8%,n:9226	mammalia_odb10	-m genome
Gene set	C:92.8%[S:90.7%,D:2.1%],F:1.5%,M:5.7%,n:12234	laurasiatheria_odb10	-m proteins
Gene set	C:92.7%[S:91.2%,D:1.5%],F:1.8%,M:5.5%,n:9226	mammalia_odb10	-m proteins

Supplementary table 3. The mapping rate of RNA-seq data of 5 organs of the raccoon dog, related to the STAR Methods.

**Organs**	**Rac 1.0 genome**	**our genome**
Heart	40.24%	95.95%
Lung	34.51%	94.54%
Spleen	52.77%	94.69%
Liver	47.89%	95.53%
Kidney	25.15%	96.28%

Supplementary table 4. Statistics of repeats in the Raccoon dog genome, related to the STAR Methods.

**Type**	**Length (bp)**	**% of genome**
Trf	59,444,898	2.50
Repeatmasker	497,544,814	20.89
Proteinmask	182,081,543	7.65
Denovo	708,412,355	29.75
Total	835,976,251	35.12

Supplementary table 5. Statistics of identified Repeats by *De novo* method in Raccoon dog genome, related to the STAR Methods.

**Type**	**Length (bp)**	**% of genome**
DNA	41,062,142	1.72
LINE	443,717,606	18.63
SINE	25,090,418	1.05
LTR	264,903,344	11.12
Satellite	5,422,754	0.23
Simple_repeat	239,342	0.01
Unknown	8,118,659	0.34
Total	708,412,355	29.75

Supplementary table 6. Transposable elements in the Raccoon dog assembly, related to the STAR Methods.

	**Repbase TEs**		**TE proteins**		***De novo***		**Combined TEs**	
**Type**	Length (bp)	% in genome	Length (bp)	% in genome	Length (bp)	% in genome	Length (bp)	% in genome
DNA	37,737,409	1.58	959,523	0.04	41,062,142	1.72	57,061,836	2.40
LINE	381,639,915	16.03	177,313,969	7.45	443,717,606	18.63	540,690,427	22.71
SINE	11,293,008	0.47	0	0.00	25,090,418	1.05	29,201,180	1.23
LTR	70,267,812	2.95	3,808,135	0.16	264,903,344	11.12	285,654,292	12.00
Other	276	0.00	0	0.00	0	0	276	0.00
Unknown	0	0.00	0	0.00	8,118,659	0.34	8,118,659	0.34
Total	497,544,814	20.89	182,081,543	7.65	702,750,259	29.51	804,603,026	33.79

Supplementary table 7. Statistics on functional annotation of the *N. procyonoides* gene set, related to the STAR Methods.

**Values**	**Total genes**	**Swissprot-Annotated**	**KEGG-Annotated**	**TrEMBL-Annotated**	**Interpro-Annotated**	**GO-Annotated**	**Overall annotated genes**
**Number**	20,000	19,665	18,027	19,794	19,910	15,099	19,973
**Percentage**	100%	98.32%	90.14%	98.97%	99.55%	75.50%	99.87%

Supplementary table 8. Statistics of ncRNA annotation, related to the STAR Methods.

Type		number	Average length (bp)	Total length (bp)	% of genome
**miRNA**		995	77.50	77,112	0.003346
**tRNA**		146,963	82.78	12,165,498	0.52791
**rRNA**	rRNA	781	170.08	132,829	0.005764
	18S	41	684.46	28,063	0.001218
	28S	267	271.27	72,430	0.003143
	5.8S	16	141.44	2263	0.000098
	5S	457	65.81	30,073	0.001305
**snRNA**	snRNA	2280	116.39	265,380	0.011516
	CD-box	330	93.67	30,911	0.001341
	HACA-box	229	137.10	31,395	0.001362
	splicing	1687	117.38	198,023	0.008593

Supplementary table 11. Genes on the X and Y chromosomes of the dog genome that mapped to the assembly in this study, related to Figure 2.

Dog genome	Chromosome	Y	X
	Accession number	NW_024010443.1<br>NW_024010444.1	NC_051843.1
	Protein-coding Genes	25	810
Mapped to our assembly	Chromosome	Scaffold30	Chr27
	Genes	25	797
	Percentage (%)	100	98.4

Supplementary table 12. The average sequencing depth (X) of the 38 raccoon dog individuals, related to Figure 2.

Sample	Sex	X	Y	Autosome
**H1**	Male	6.29	5.27	12.2552
**H2**	Male	7.45	5.94	13.7322
**H3**	Female	10.3	0.1	11.2975
**H4**	Male	8.04	6.26	14.1528
**H5**	Male	7.79	6.28	14.1281
**H6**	Male	7.12	5.56	12.5209
**H7**	Male	5.69	4.24	10.8209
**H8**	Male	7.46	6.1	13.5387
**H9**	Male	7.27	5.9	13.1706
**H10**	Male	7.34	5.85	14.0819
**H11**	Male	7.14	5.71	13.2633
**H12**	Male	7.18	5.27	12.4231
**H13**	Male	6.9	5.5	12.0514
**H14**	Male	6.47	5.05	11.4562
**H15**	Male	6.84	5.37	12.5308
**H16**	Male	7.58	5.99	12.8319
**H17**	Male	7.02	5.42	12.6984
**H18**	Male	7.29	5.19	12.6699
**H19**	Male	6.94	4.97	12.7548
**H20**	Male	6.66	4.84	12.3026

**H21**	Male	6.71	5.17	12.9543
**H22**	Male	6.69	5.28	12.6165
**H23**	Female	10.43	0.04	11.9991
**H24**	Male	6.09	4.21	10.4629
**H25**	Male	7.47	5.71	12.9038
**H26**	Male	7.57	5.9	12.9395
**H27**	Male	6.82	4.95	12.3355
**H29**	Male	8.49	6.99	14.2662
**H30**	Female	13.62	0.04	13.858
**H31**	Male	8.39	6.6	14.3209
**H32**	Male	7.99	6.45	14.2982
**H33**	Male	8.03	6.52	14.4977
**H34**	Female	11.18	0.05	11.7251
**H35**	Female	10.8	0.02	11.8172
**H36**	Male	8.21	7.12	15.5009
**H37**	Male	7.77	6.76	13.8287
**H38**	Male	6.07	4.6	11.4898
**H39**	Male	6.87	5.55	13.0006

Supplementary table 13. Species used in comparative genomics analysis, related to Figure 3A.

**Species**	**Short Name**	**Database**	**Version**
Bos taurus	*B. taurus*	Ensembl	ARS-UCD1.2
Ursus thibetanus thibetanus	*U. thibetanus thibetanus*	Ensembl	ASM966005v1
Acinonyx jubatus	*A. jubatus*	NCBI	GCF_003709585.1_Aci_jub_2
Ursus arctos	*U. arctos*	NCBI	GCF_003584765.1_ASM358476v1
Sus scrofa	*S. scrofa*	Ensembl	Sscrofa11.1
Panthera leo	*P. leo*	Ensembl	PanLeo1.0
Panthera pardus	*P. pardus*	Ensembl	PanPar1.0
Nyctereutes procyonoides	*N. procyonoides*	--	this study
Vulpes lagopus	*V. lagopus*	NCBI	GCF_018345385.1_ASM1834538v1
Ursus maritimus	*U. maritimus*	Ensembl	UrsMar_1.0
Canis lupus dingo	*C. lupus dingo*	Ensembl	ASM325472v1
Panthera tigris altaica	*P. tigris altaica*	Ensembl	PanTig1.0
Vulpes vulpes	*V. vulpes*	Ensembl	VulVul2.2
Homo sapiens	*H. sapiens*	Ensembl	GRCh38
Felis catus	*F. catus*	Ensembl	Felis_catus_9.0
Equus caballus	*E. caballus*	Ensembl	EquCab3.0
Canis lupus familiaris	*C. lupus familiaris*	Ensembl	CanFam3.1
Oryctolagus cuniculus	*O. cuniculus*	Ensembl	OryCun2.0

Supplementary table 15. The KEGG enrichment result of expanded families in the raccoon dog genome compared with 17 species, related to the STAR Methods.

MapID	MapTitle	*p* value	Adjusted *p* value	Number
map03410	Base excision repair	2.12E-74	1.22E-72	124
map04217	Necroptosis	5.17E-58	2.47E-56	181
map03040	Spliceosome	2.29E-21	3.65E-20	121
map03050	Proteasome	1.46E-07	1.13E-06	39
map00010	Glycolysis / Gluconeogenesis	4.62E-46	1.33E-44	100
map01200	Carbon metabolism	4.37E-35	8.95E-34	115
map00620	Pyruvate metabolism	1.63E-13	1.95E-12	40
map04714	Thermogenesis	7.58E-11	8.36E-10	121
map00020	Citrate cycle (TCA cycle)	4.87E-05	0.000176	21
map00640	Propanoate metabolism	0.000504402	0.001379	21
map04066	HIF-1 signaling pathway	3.33E-39	7.96E-38	122
map00190	Oxidative phosphorylation	5.03E-19	7.21E-18	100
map04740	Olfactory transduction	1.31E-05	5.79E-05	112
map00982	Drug metabolism - cytochrome P450	0.001375309	0.003461	24
map00270	Cysteine and methionine metabolism	1.16E-20	1.76E-19	55
map00480	Glutathione metabolism	0.003440545	0.007596	26
map04970	Salivary secretion	1.35E-40	3.52E-39	118
map05012	Parkinson disease	6.26E-15	8.17E-14	93
map05130	Pathogenic Escherichia coli infection	1.11E-12	1.27E-11	117
map04216	Ferroptosis	3.82E-07	2.55E-06	31
map05212	Pancreatic cancer	0.000116986	0.000373	40
map05322	Systemic lupus erythematosus	0.003917862	0.008454	43
map05210	Colorectal cancer	0.004171609	0.008869	38
map03008	Ribosome biogenesis in eukaryotes	0.015976408	0.02813	34
map05016	Huntington disease	6.09E-51	2.50E-49	198
map05140	Leishmaniasis	4.46E-48	1.60E-46	120
map04212	Longevity regulating pathway - worm	1.47E-47	4.68E-46	126
map03013	RNA transport	2.80E-37	6.17E-36	157
map05010	Alzheimer disease	1.00E-31	1.80E-30	151
map04210	Apoptosis	1.33E-29	2.25E-28	129
map05134	Legionellosis	5.10E-101	7.32E-99	159
map00532	Glycosaminoglycan biosynthesis - chondroitin sulfate / dermatan sulfate	1.12E-89	1.08E-87	115
map04214	Apoptosis - fly	4.22E-76	3.03E-74	132
map05230	Central carbon metabolism in cancer	0.002551893	0.005859	28
map00630	Glyoxylate and dicarboxylate metabolism	1.22E-06	7.03E-06	25
map01230	Biosynthesis of amino acids	8.07E-34	1.54E-32	91
map03010	Ribosome	0	0	527

Supplementary table 16. Prediction of taste receptor genes in raccoon dog genome, related to the STAR Methods.

Protein	Annotated Gene
NPRO_08429	*TAS1R1*
NPRO_10341-D1	*TAS1R2*
NPRO_08491	*TAS1R3*
NPRO_00947	TAS2R gene family
NPRO_00948	
NPRO_00950	
NPRO_00951	
NPRO_00952	
NPRO_00954	
NPRO_00955-D1	
NPRO_00956	
NPRO_01202	
NPRO_01210	
NPRO_01211	
NPRO_01235	
NPRO_01239	
NPRO_01240	
NPRO_08825	
NPRO_17195	

Supplementary table 19. Summary of functions of immune-related genes that under positive selection, related to the STAR Methods.

**Gene**	**Function**	**Reference**
ACER3	mediates the immune response in cells of the innate immune system; anti-inflammatory function	(Wang et al., 2016)
AKAP1	involved in antiviral innate immunity mediated by mitochondria	(Yoshinaka et al., 2019)
AXIN1	preventive effect on bacterial invasion and inflammatory response in the early stage of infection	(Zhang et al., 2012)
CCL20	immune modulator in both adaptive and innate immune responses	(Ranasinghe and Eri, 2018)
CD99L2	involved in the inflammatory response as an adhesion molecule	(Schenkel et al., 2007)
CDH17	plays important roles in early and late B cell development	(Funakoshi et al., 2015)
CSF3	regulates immune response to pathogens, promote leukocyte proliferation, migration, and phagocytosis	(Li et al., 2021)
EZH1	regulates innate immune response by involving in cytokines production triggered by TLR	(Liu et al., 2015)
FGD2	involved in leukocyte signaling and presenting antigen in the immune system	(Huber et al., 2008)
GRIP1	a critical regulator of immunometabolism to coordinate the balance between macrophage populations and ultimately promote metabolic homeostasis	(Coppo et al., 2016)
HERC6	antiviral innate immunity	(Jacquet et al., 2020)
HIF1A	many functions in both innate and adaptive immunity	(Palazon et al., 2014)

IGF1	an important switch to govern the amplitude and quality of immune responses	(Smith, 2010)
IKBKB	activates NF-κB signaling. Mutations in the *IKBKB* gene cause severe immunodeficiency	(Qin et al., 2020)
IL25	plays a role in differentiation of Th2 and Th2 memory cells	(Valizadeh et al., 2015)
IL5	involved in a number of immune responses; plays roles in innate immunity by maintaining B-1 B cells and mucosal IgA production	(Ikutani et al., 2012)
ITGB2	plays a role in T-cell development and function	(Moore et al., 2008)
MALT1	a central signaling component in both innate and adaptive *immunity* by regulating NF-κB pathway	(Demeyer et al., 2019)
NEDD9	plays an important role in chemotaxis, cell adhesion and lymphocyte movement, influencing functions of both B and T lymphocytes	(Seo et al., 2005)
NLRX1	critical in regulating pathogen response and plays an important role in innate immunity	(Pickering and Booty, 2021)
OTUD5	critical for effective innate antiviral	(Guo et al., 2021)
PEX1	regulation of the c-Fos mediated pathway in innate immunity	(Murphy, 2020)
PRKCD	an essential regulator of peripheral B-cell development and a critical regulator of immune homeostasis	(Salzer et al., 2016)
RNF216	RNF216 has been implicated in regulating innate immunity signaling pathways	(Cotton et al., 2022)
SMC4	enhances inflammatory innate immunity	(Wang et al., 2018)
SNX25	inhibits the NF-κB signal to regulate expression of proinflammatory cytokine in macrophages	(Nishimura et al., 2021)
STAT4	a transcription factor that transduces interleukin-23, interleukin-12, and type I interferon cytokine signals in monocytes and T cells, leading to T-helper type 17 and T-helper type 1 differentiation	(Korman et al., 2008)
TEC	mature T-cell differentiation and T-cell development	(Readinger et al., 2009)
TRPV2	multiple functions in innate and adaptive immunity	(Santoni et al., 2013)
TYK2	involved in various processes in innate and adaptive immunity	(Strobl et al., 2011)

Supplementary table 20. Raccoon dog specific amino acid changes compared with human and other canids, related to the STAR Methods.

**Gene**	**Mutation**	**Domain**	**Domain position**
AKAP1	K129E	--	--
AKAP1	P137L	--	--
AKAP1	P281S	--	--
AXIN1	A443T	--	--
AXIN1	G455S	--	--
CCL20	A28S	--	--
CCL20	P44A	Chemokine super family	31-91
CDH17	P553V	Cadherin tandem repeat domain	491-597
CSF3	A78T	IL6 super family	40-194
EZH1	A415H	--	--
FGD2	D280N	RhoGEF domain	106-290

HERC6	P68Q	--	--
HERC6	G42R	ATS1 super family	25-282
HERC6	L944W	HECT-domain	660-1002
HERC6	P182L	ATS1 super family	25-282
HIF1A	N416I	--	--
IKBKB	V548M	IQBAL scaffold dimerization domain	330-604
IL25	R80S	Interleukin-17	74-160
NLRX1	L350M	--	--
PRKCD	K667V	Protein Kinases, catalytic domain	395-710
RNF216	D250N	--	--
RNF216	Q209H	--	--
SMC4	F617L	SMC_N domain	120-1311
SMC4	H675Q	SMC_N domain	120-1311
SMC4	Q587H	SMC_N domain	120-1311
SMC4	R549A	SMC_N domain	120-1311
SMC4	K474Q	SMC_N domain	120-1311
SMC4	R815G	SMC_N domain	120-1311
SNX25	Q935R	PX_SNX25 domain	665-787
SNX25	N492V	RGS_SNX25 domain	454-562
SNX25	E170V	PXA domain	165-320
STAT4	S512T	--	--
STAT4	P544Y	--	--
TRPV2	G565E	TRPV2 domain	70-712
TRPV2	E585S	TRPV3 domain	70-712
TDRD6	D724N	--	--
TDRD6	Q963E	--	--
TDRD6	N793D	Tudor domain superfamily	767-886
ZP3	H143L	--	--
ZP3	S144V	ZP domain	44-300
ZP3	D315G	--	--

Supplementary table 21. The frequency of raccoon dog specific amino acid changes in the population level, related to the STAR Methods.

**GENE**	**Mutation**	**Amino acid**	**Frequency in population**	**Alternative Amino acid**	**Frequency in population**
AKAP1	E129K	E	1	--	0
AKAP1	L137P	L	1	--	0
AKAP1	S281P	S	1	--	0
AXIN1	T443A	T	1	--	0
AXIN1	S455G	S	1	--	0
CCL20	S28A	S	0.05	A	0.95
CCL20	A44P	A	1	--	0
CDH17	V553P	V	1	--	0
CSF3	T78A	T	1	--	0
EZH1	H415A	H	1	--	0
FGD2	N280D	N	0.55	D	0.45
HERC6	R42G	R	1	--	0

HERC6	Q68P	Q	1	--	0
HERC6	L182P	L	1	--	0
HERC6	W944L	W	1	--	0
HIF1A	I416N	I	1	--	0
IKBKB	M548V	M	1	--	0
IL25	S80R	S	1	--	0
NLRX1	M350L	M	1	--	0
PRKCD	V667K		1	--	0
RNF216	H209Q	H	1	--	0
RNF216	N250D	N	1	--	0
SMC4	Q474K	Q	1	--	0
SMC4	A549R	A	1	--	0
SMC4	H587Q	H	1	--	0
SMC4	L617F	L	1	--	0
SMC4	Q675H	Q	1	--	0
SMC4	G815R	G	1	--	0
SNX25	V170E	V	1	--	0
SNX25	V492N	V	1	--	0
SNX25	R935Q	R	1	--	0
STAT4	T512S	T	0.97	--	0.03
STAT4	Y544P	Y	1	--	0
TRPV2	E565G	E	0.14	G	0.86
TRPV2	S585E	S	1	--	0
TDRD6	N724D	N	1	--	0
TDRD6	D793N	D	1	--	0
TDRD6	E963Q	E	1	--	0
ZP3	L143H	L	1	--	0
ZP3	V144S	V	1	--	0
ZP3	G315D	G	1	--	0

Supplementary table 22. Summary of tumor suppressor genes that under positive selection, related to the STAR Methods.

**Gene**	**Cancer**	**Reference**
RERG	brest cancer, nasopharyngeal carcinoma	(Zhao et al., 2017)
BRCA1	brest cancer, ovarian cancer	(Silver and Livingston, 2012)
FETUB	prostate cancer	(Zhan et al., 2020)
RNF20	breast cancer	(Shema et al., 2008)
MYO18B	lung cancer	(Nishioka et al., 2002)
RBM5	lung cancer	(Jamsai et al., 2017)
BMP3	colon cancer, biliary cancers	(Kisiel et al., 2013)
LACTB	breast cancer	(Keckesova et al., 2017)
UNC5A	lung cancer, bladder cancer	(Ding et al., 2020)
PCK1	liver tumor	(Tuo et al., 2019)

Supplementary table 23. Summary of energy metabolism related genes that under positive selection, related to the STAR Methods.

Gene	Function	Reference
CDKAL1	required for normal mitochondrial function and morphology	(Palmer et al., 2017)
TRIM63	energy and maintains energy homeostasis in heart	(Peris-Moreno et al., 2020)
GALNT13	involved in metabolism and energy pathways	(Maciejewska-Skrendo et al., 2019)
PRKAG3	plays a key role in energy metabolism in skeletal muscle	(Ryan et al., 2012)
RPUSD4	OXPHOS homeostasis, regulates the mitochondrial 16S rRNA and intra-mitochondrial translation	(Arroyo et al., 2016)
MRPL19	a central role in ribosome mitochondrial protein synthesis, minor changes in the protein lead to impaired energy metabolism	(Anthoni et al., 2007)

Supplementary table 24. The non-hibernating animals used for comparative genomic analysis with the raccoon dog, related to the STAR Methods.

Species	Short Name	Database	Version
Bos taurus	*B. taurus*	Ensembl	ARS-UCD1.2
Acinonyx jubatus	*A. jubatus*	NCBI	GCF_003709585.1_Aci_jub_2
Sus scrofa	*S. scrofa*	Ensembl	Sscrofa11.1
Panthera leo	*P. leo*	Ensembl	PanLeo1.0
Panthera pardus	*P. pardus*	Ensembl	PanPar1.0
Vulpes lagopus	*V. lagopus*	NCBI	GCF_018345385.1_ASM1834538v1
Canis lupus dingo	*C. lupus dingo*	Ensembl	ASM325472v1
Panthera tigris altaica	*P. tigris altaica*	Ensembl	PanTig1.0
Vulpes vulpes	*V. vulpes*	Ensembl	VulVul2.2
Homo sapiens	*H. sapiens*	Ensembl	GRCh38
Felis catus	*F. catus*	Ensembl	Felis_catus_9.0
Equus caballus	*E. caballus*	Ensembl	EquCab3.0
Canis lupus familiaris	*C. lupus familiaris*	Ensembl	CanFam3.1
Oryctolagus cuniculus	*O. cuniculus*	Ensembl	OryCun2.0

Supplementary table 26. Summary of functions of immune-related genes that under positive selection in the raccoon dog genome by comparing with 14 other non-hibernating animals, related to STAR Methods.

Gene	Function	Reference
CCL20	immune modulator in both adaptive and innate immune responses	(Ranasinghe and Eri, 2018)
DNMT3B	DNMT3B deficiency may present as severe combined immune deficiency (SCID)	(Mehawej et al., 2020)
FER	electroporation-mediated delivery of FER gene enhances innate immune response	(Dolgachev et al., 2018)
GRIP1	a critical regulator of immunometabolism to coordinate the balance between macrophage populations and ultimately promote metabolic homeostasis	(Coppo *et al.*, 2016)
HERC6	antiviral innate immunity	(Jacquet *et al.*, 2020)
HIF1A	many functions in both innate and adaptive immunity	(Palazon *et al.*, 2014)
IL13RA1	Essential for Induction of the Alternative Macrophage Activation Pathway by IL-13	(Sheikh et al., 2015)
IKBKB	activates NF-κB signaling. Mutations in the *IKBKB* gene cause severe immunodeficiency	(Qin *et al.*, 2020)
IL5	involved in a number of immune responses; plays roles in innate immunity by maintaining B-1 B cells and mucosal IgA production	(Ikutani *et al.*, 2012)
ITGB2	plays a role in T-cell development and function	(Moore *et al.*, 2008)
LRP1	LRP1 modulates the microglial immune response via regulation of JNK and NF-κB signaling pathways	(Yang et al., 2016)
MANBA	The NFKB1/MANBA gene region play important role in regulating the immune system	(Hitomi et al., 2019)
NFAT5	NFAT5 plays a role in the development and activation of immune cells, especially T cells and macrophages	(Lee et al., 2019)
NLRX1	critical in regulating pathogen response and plays an important role in innate immunity	(Pickering and Booty, 2021)
PLD1	PLD1 deficiency impaired TCR-mediated signaling, T cell expansion, and effector function during immune responses against Listeria monocytogene	(Zhu et al., 2018)
RNF216	RNF216 has been implicated in regulating innate immunity signaling pathways	(Cotton *et al.*, 2022)
SMC4	enhances inflammatory innate immunity	(Wang *et al.*, 2018)
STAT4	a transcription factor that transduces interleukin-23, interleukin-12, and type I interferon cytokine signals in monocytes and T cells, leading to T-helper type 17 and T-helper type 1 differentiation	(Korman *et al.*, 2008)
TEC	mature T-cell differentiation and T-cell development	(Readinger *et al.*, 2009)
TRPV2	multiple functions in innate and adaptive immunity	(Santoni *et al.*, 2013)
TYK2	involved in various processes in innate and adaptive immunity	(Strobl *et al.*, 2011)

Supplementary table 27. Summary of energy metabolism related genes that under positive selection in the raccoon dog genome by comparing with 14 other non-hibernating animals, related to STAR Methods.

Gene	Function	Reference
AOC1	important for cellular energy metabolism and mitochondrial iron transport	(Kallianpur et al., 2014)

CDKAL1	required for normal mitochondrial function and morphology	(Palmer *et al.*, 2017)
COX11	Involved in glucose metabolism and energy he homeostasis	(Aslanukov et al., 2005)
MRPL19	participate in mitochondrial energy metabolism, minor changes in the protein, leading to marginally impaired energy metabolism may have developmental consequences in critical tissues.	(Anthoni *et al.*, 2007)
PCK1	Key enzyme in the gluconeogenesis pathway	(Tuo *et al.*, 2019)

Supplementary table 28. Summary of feeding behavior, insulin resistance, and lipid metabolism related genes that under positive selection in the raccoon dog genome by comparing with 14 other non-hibernating animals, related to STAR Methods.

**Gene**	**Function**	**Reference**
HCRTR1	the receptor gene of orexin, which is involved in foraging behavior and intake of energy-dense food	(Barson, 2020)
GOLGB1	plays crucial role in the development of the mammalian palate	(Lan et al., 2016)
MLN	regulates gastrointestinal contractions and stimulates hunger signaling	(Tack et al., 2016)
STXBP5L	a negative regulator of insulin secretion	(Bhatnagar et al., 2011)
SEC16B	modulates obesity by controlling dietary lipid absorption	(Shi et al., 2021)
BSCL2	regulates cAMP/PKA-mediated lipolysis, which is essential for white adipocyte differentiation and maintenance; Deletion of BSCL2 in mature white and brown adipose tissue also triggers cAMP/PKA-mediated lipolysis and FAO resulting in adipose tissue loss.	(Zhou et al., 2022; Zhou et al., 2020)

Supplementary table 29. The genome-wide heterozygosity of the raccoon dog and other 17 other species, related to Figure 5A.

**Species**	**Heterozygosity (%)**	**Sources**
Iberian lynx (*Lynx pardinus*)	0.01	(Abascal et al., 2016)
Domestic cat (*Felis catus*)	0.012	(Cho et al., 2013)
Cheetah (*Acinonyx jubatus*)	0.020	(Dobrynin et al., 2015)
Snow leopard (*Panthera uncia syn*)	0.023	(Cho *et al.*, 2013)
Eurasian lynx (Abascal *et al.*)	0.028	(Abascal *et al.*, 2016)
Domestic dog (*Canis familiaris*)	0.032	(Lindblad-Toh et al., 2005)
Amur tiger (*Panthera tigris altaica*)	0.049	(Cho *et al.*, 2013)
African lion (*Panthera leo*)	0.058	(Cho *et al.*, 2013)
Gray fox (*Urocyon cinereoargenteus*)	0.120	(Robinson et al., 2016)
Rat (*Rattus norvegicus*)	0.125	(Leffler et al., 2012)
Gray wolf (*Canis lupus*)	0.149	(Corbett-Detig et al., 2015)
Raccoon dog (*Nyctereutes procyonoides*)	0.28	This study
Kinkajou (*Potos flavus*)	0.35	(Tsuchiya et al., 2021)
Raccoon (*Procyon lotor*)	0.44	(Tsuchiya *et al.*, 2021)

Wild boar (*Sus scrofa*)	0.441	(Corbett-Detig *et al.*, 2015)
Black flying fox (*Pteropus alecto*)	0.453	(Zhang et al., 2013)
Opossum (*Monodelphis domestica*)	0.490	(Mikkelsen et al., 2007)
Marbled crayfish (*Procambarus virginalis*)	0.53	(Gutekunst et al., 2018)

References

Abascal, F., Corvelo, A., Cruz, F., Villanueva-Cañas, J.L., Vlasova, A., Marcet-Houben, M., Martínez-Cruz, B., Cheng, J.Y., Prieto, P., and Quesada, V. (2016). Extreme genomic erosion after recurrent demographic bottlenecks in the highly endangered Iberian lynx. Genome biology 17, 1-19.

Anthoni, H., Zucchelli, M., Matsson, H., Muller-Myhsok, B., Fransson, I., Schumacher, J., Massinen, S., Onkamo, P., Warnke, A., Griesemann, H., et al. (2007). A locus on 2p12 containing the co-regulated MRPL19 and C2ORF3 genes is associated to dyslexia. Human molecular genetics 16, 667-677. 10.1093/hmg/ddm009.

Arroyo, J.D., Jourdain, A.A., Calvo, S.E., Ballarano, C.A., Doench, J.G., Root, D.E., and Mootha, V.K. (2016). A Genome-wide CRISPR Death Screen Identifies Genes Essential for Oxidative Phosphorylation. Cell metabolism 24, 875-885. 10.1016/j.cmet.2016.08.017.

Aslanukov, A., Bhowmick, R., Guruju, M., Oswald, J., Raz, D., Bush, R.A., Sieving, P., Lu, X., Bock, C.B., and Ferreira, P.A. (2005). RanBP2 Modulates Cox11 and Hexokinase I Activities and Haploinsufficiency of RanBP2 Causes Deficits in Hexokinase I and Glucose Metabolism. PLoS genetics preprint, e177. 10.1371/journal.pgen.0020177.eor.

Barson, J.R. (2020). Orexin/hypocretin and dysregulated eating: Promotion of foraging behavior. Brain research 1731, 145915. 10.1016/j.brainres.2018.08.018.

Bhatnagar, S., Oler, A.T., Rabaglia, M.E., Stapleton, D.S., Schueler, K.L., Truchan, N.A., Worzella, S.L., Stoehr, J.P., Clee, S.M., Yandell, B.S., et al. (2011). Positional cloning of a type 2 diabetes quantitative trait locus; tomosyn-2, a negative regulator of insulin secretion. PLoS genetics 7, e1002323. 10.1371/journal.pgen.1002323.

Cho, Y.S., Hu, L., Hou, H., Lee, H., Xu, J., Kwon, S., Oh, S., Kim, H.-M., Jho, S., and Kim, S. (2013). The tiger genome and comparative analysis with lion and snow leopard genomes. Nature communications 4, 1-7.

Coppo, M., Chinenov, Y., Sacta, M.A., and Rogatsky, I. (2016). The transcriptional coregulator GRIP1 controls macrophage polarization and metabolic homeostasis. Nature communications 7, 12254. 10.1038/ncomms12254.

Corbett-Detig, R.B., Hartl, D.L., and Sackton, T.B. (2015). Natural selection constrains neutral diversity across a wide range of species. PLoS biology 13, e1002112.

Cotton, T.R., Cobbold, S.A., Bernardini, J.P., Richardson, L.W., Wang, X.S., and Lechtenberg, B.C. (2022). Structural basis of K63-ubiquitin chain formation by the Gordon-Holmes syndrome RBR E3 ubiquitin ligase RNF216. Molecular cell 82, 598-615 e598. 10.1016/j.molcel.2021.12.005.

Demeyer, A., Skordos, I., Driege, Y., Kreike, M., Hochepied, T., Baens, M., Staal, J., and Beyaert, R. (2019). MALT1 Proteolytic Activity Suppresses Autoimmunity in a T Cell Intrinsic Manner. Frontiers in immunology 10, 1898. 10.3389/fimmu.2019.01898.

Ding, S., Zhang, H., Zhao, X., Dang, J., and Li, G. (2020). UNC5A, an epigenetically silenced gene, functions as a tumor suppressor in non-small cell lung cancer. Saudi journal of biological sciences 27, 3009-3017. 10.1016/j.sjbs.2020.09.023.

Dobrynin, P., Liu, S., Tamazian, G., Xiong, Z., Yurchenko, A.A., Krasheninnikova, K., Kliver, S., Schmidt-Küntzel, A., Koepfli, K.-P., and Johnson, W. (2015). Genomic legacy of the African cheetah, Acinonyx jubatus. Genome biology 16, 1-20.

Dolgachev, V., Panicker, S., Balijepalli, S., McCandless, L.K., Yin, Y., Swamy, S., Suresh, M.V., Delano, M.J., Hemmila, M.R., Raghavendran, K., and Machado-Aranda, D. (2018). Electroporation-mediated delivery of FER gene enhances innate immune response and improves survival in a murine model of pneumonia. Gene therapy 25, 359-375. 10.1038/s41434-018-0022-y.

Funakoshi, S., Shimizu, T., Numata, O., Ato, M., Melchers, F., and Ohnishi, K. (2015). BILL-cadherin/cadherin-17 contributes to the survival of memory B cells. PloS one 10, e0117566. 10.1371/journal.pone.0117566.

Guo, Y., Jiang, F., Kong, L., Wu, H., Zhang, H., Chen, X., Zhao, J., Cai, B., Li, Y., Ma, C., et al. (2021). OTUD5 promotes innate antiviral and antitumor immunity through deubiquitinating and stabilizing STING. Cellular & molecular immunology 18, 1945-1955. 10.1038/s41423-020-00531-5.

Gutekunst, J., Andriantsoa, R., Falckenhayn, C., Hanna, K., Stein, W., Rasamy, J., and Lyko, F. (2018). Clonal genome evolution and rapid invasive spread of the marbled crayfish. Nat Ecol Evol 2, 567-573. 10.1038/s41559-018-0467-9.

Hitomi, Y., Nakatani, K., Kojima, K., Nishida, N., Kawai, Y., Kawashima, M., Aiba, Y., Nagasaki, M., Nakamura, M., and Tokunaga, K. (2019). NFKB1 and MANBA Confer Disease Susceptibility to Primary Biliary Cholangitis via Independent Putative Primary Functional Variants. Cellular and molecular gastroenterology and hepatology 7, 515-532. 10.1016/j.jcmgh.2018.11.006.

Huber, C., Martensson, A., Bokoch, G.M., Nemazee, D., and Gavin, A.L. (2008). FGD2, a CDC42-specific exchange factor expressed by antigen-presenting cells, localizes to early endosomes and active membrane ruffles. The Journal of biological chemistry 283, 34002-34012. 10.1074/jbc.M803957200.

Ikutani, M., Yanagibashi, T., Ogasawara, M., Tsuneyama, K., Yamamoto, S., Hattori, Y., Kouro, T., Itakura, A., Nagai, Y., Takaki, S., and Takatsu, K. (2012). Identification of innate IL-5-producing cells and their role in lung eosinophil regulation and antitumor immunity. Journal of immunology 188, 703-713. 10.4049/jimmunol.1101270.

Jacquet, S., Pontier, D., and Etienne, L. (2020). Rapid Evolution of HERC6 and Duplication of a Chimeric HERC5/6 Gene in Rodents and Bats Suggest an Overlooked Role of HERCs in Mammalian Immunity. Frontiers in immunology 11, 605270. 10.3389/fimmu.2020.605270.

Jamsai, D., Watkins, D.N., O'Connor, A.E., Merriner, D.J., Gursoy, S., Bird, A.D., Kumar, B., Miller, A., Cole, T.J., Jenkins, B.J., and O'Bryan, M.K. (2017). In vivo evidence that RBM5 is a tumour suppressor in the lung. Scientific reports 7, 16323. 10.1038/s41598-017-15874-9.

Kallianpur, A.R., Jia, P., Ellis, R.J., Zhao, Z., Bloss, C., Wen, W., Marra, C.M., Hulgan, T., Simpson, D.M., Morgello, S., et al. (2014). Genetic variation in iron metabolism is associated with neuropathic pain and pain severity in HIV-infected patients on antiretroviral therapy. PloS one 9, e103123. 10.1371/journal.pone.0103123.

Keckesova, Z., Donaher, J.L., De Cock, J., Freinkman, E., Lingrell, S., Bachovchin, D.A., Bierie, B., Tischler, V., Noske, A., Okondo, M.C., et al. (2017). LACTB is a tumour suppressor that modulates lipid metabolism and cell state. Nature 543, 681-686. 10.1038/nature21408.

Kisiel, J.B., Li, J., Zou, H., Oseini, A.M., Strauss, B.B., Gulaid, K.H., Moser, C.D., Aderca, I., Ahlquist, D.A., Roberts, L.R., and Shire, A.M. (2013). Methylated Bone Morphogenetic Protein 3 (BMP3) Gene: Evaluation of Tumor Suppressor Function and Biomarker Potential in Biliary Cancer. Journal of molecular biomarkers & diagnosis 4, 1000145. 10.4172/2155-9929.1000145.

Korman, B.D., Kastner, D.L., Gregersen, P.K., and Remmers, E.F. (2008). STAT4: genetics, mechanisms, and implications for autoimmunity. Current allergy and asthma reports 8, 398-403. 10.1007/s11882-008-0077-8.

Lan, Y., Zhang, N., Liu, H., Xu, J., and Jiang, R. (2016). Golgb1 regulates protein glycosylation and is crucial for mammalian palate development. Development 143, 2344-2355. 10.1242/dev.134577.

Lee, N., Kim, D., and Kim, W.U. (2019). Role of NFAT5 in the Immune System and Pathogenesis of Autoimmune Diseases. Frontiers in immunology 10, 270. 10.3389/fimmu.2019.00270.

Leffler, E.M., Bullaughey, K., Matute, D.R., Meyer, W.K., Segurel, L., Venkat, A., Andolfatto, P., and Przeworski, M. (2012). Revisiting an old riddle: what determines genetic diversity levels within species?

Li, W., Guan, X., Sun, B., and Sun, L. (2021). A Novel microRNA of Japanese Flounder Regulates Antimicrobial Immunity Involving a Bacteria-Binding CSF3. Frontiers in immunology 12, 723401. 10.3389/fimmu.2021.723401.

Lindblad-Toh, K., Wade, C.M., Mikkelsen, T.S., Karlsson, E.K., Jaffe, D.B., Kamal, M., Clamp, M., Chang, J.L., Kulbokas, E.J., and Zody, M.C. (2005). Genome sequence, comparative analysis and haplotype structure of the domestic dog. Nature 438, 803-819.

Liu, Y., Zhang, Q., Ding, Y., Li, X., Zhao, D., Zhao, K., Guo, Z., and Cao, X. (2015). Histone lysine methyltransferase Ezh1 promotes TLR-triggered inflammatory cytokine production by suppressing Tollip. Journal of immunology 194, 2838-2846. 10.4049/jimmunol.1402087.

Maciejewska-Skrendo, A., Cieszczyk, P., Chycki, J., Sawczuk, M., and Smolka, W. (2019). Genetic Markers Associated with Power Athlete Status. Journal of human kinetics 68, 17-36. 10.2478/hukin-2019-0053.

Mehawej, C., Khalife, H., Hanna-Wakim, R., Dbaibo, G., and Farra, C. (2020). DNMT3B deficiency presenting as severe combined immune deficiency: A case report. Clinical immunology 215, 108453. 10.1016/j.clim.2020.108453.

Mikkelsen, T.S., Wakefield, M.J., Aken, B., Amemiya, C.T., Chang, J.L., Duke, S., Garber, M., Gentles, A.J., Goodstadt, L., and Heger, A. (2007). Genome of the marsupial Monodelphis domestica reveals innovation in non-coding sequences. Nature 447, 167-177.

Moore, S.W., Sidler, D., and Zaahl, M.G. (2008). The ITGB2 immunomodulatory gene (CD18), enterocolitis, and Hirschsprung's disease. Journal of pediatric surgery 43, 1439-1444. 10.1016/j.jpedsurg.2007.12.057.

Murphy, S. (2020). PEX1 and PEX7 Genes are Necessary for Completion of Immune Pathways and Survival Post-Infection. STEM Fellowship Journal 6, 7.

Nishimura, K., Tanaka, T., Takemura, S., Tatsumi, K., and Wanaka, A. (2021). SNX25 regulates proinflammatory cytokine expression via the NF-kappaB signal in macrophages. PloS one 16, e0247840. 10.1371/journal.pone.0247840.

Nishioka, M., Kohno, T., Tani, M., Yanaihara, N., Tomizawa, Y., Otsuka, A., Sasaki, S., Kobayashi, K., Niki, T., Maeshima, A., et al. (2002). MYO18B, a candidate tumor suppressor gene at chromosome 22q12.1, deleted, mutated, and methylated in human lung cancer. Proceedings of the National Academy of Sciences of the United States of America 99, 12269-12274. 10.1073/pnas.192445899.

Palazon, A., Goldrath, A.W., Nizet, V., and Johnson, R.S. (2014). HIF transcription factors, inflammation, and immunity. Immunity 41, 518-528. 10.1016/j.immuni.2014.09.008.

Palmer, C.J., Bruckner, R.J., Paulo, J.A., Kazak, L., Long, J.Z., Mina, A.I., Deng, Z., LeClair, K.B., Hall, J.A., Hong, S., et al. (2017). Cdkal1, a type 2 diabetes susceptibility gene, regulates mitochondrial function in adipose tissue. Molecular metabolism 6, 1212-1225. 10.1016/j.molmet.2017.07.013.

Peris-Moreno, D., Taillandier, D., and Polge, C. (2020). MuRF1/TRIM63, Master Regulator of Muscle Mass. International journal of molecular sciences 21. 10.3390/ijms21186663.

Pickering, R.J., and Booty, L.M. (2021). NLR in eXile: Emerging roles of NLRX1 in immunity and human disease. Immunology 162, 268-280. 10.1111/imm.13291.

Qin, T., Jia, Y., Liu, Y., Dai, R., Zhou, L., Okada, S., Tsumura, M., Ohnishi, H., Kato, Z., Kanegane, H., et al. (2020). A Novel Homozygous Mutation Destabilizes IKKbeta and Leads to Human Combined Immunodeficiency. Frontiers in immunology 11, 517544. 10.3389/fimmu.2020.517544.

Ranasinghe, R., and Eri, R. (2018). CCR6–CCL20-Mediated Immunologic Pathways in Inflammatory Bowel Disease. Gastrointestinal Disorders 1, 15-29. 10.3390/gidisord1010003.

Readinger, J.A., Mueller, K.L., Venegas, A.M., Horai, R., and Schwartzberg, P.L. (2009). Tec kinases regulate T-lymphocyte development and function: new insights into the roles of Itk and Rlk/Txk. Immunological reviews 228, 93-114. 10.1111/j.1600-065X.2008.00757.x.

Robinson, J.A., Ortega-Del Vecchyo, D., Fan, Z., Kim, B.Y., Marsden, C.D., Lohmueller, K.E., and Wayne, R.K. (2016). Genomic flatlining in the endangered island fox. Current Biology 26, 1183-1189.

Ryan, M.T., Hamill, R.M., O'Halloran, A.M., Davey, G.C., McBryan, J., Mullen, A.M., McGee, C., Gispert, M., Southwood, O.I., and Sweeney, T. (2012). SNP variation in the promoter of the PRKAG3 gene and association with meat quality traits in pig. BMC genetics 13, 66. 10.1186/1471-2156-13-66.

Salzer, E., Santos-Valente, E., Keller, B., Warnatz, K., and Boztug, K. (2016). Protein Kinase C delta: a Gatekeeper of Immune Homeostasis. Journal of clinical immunology 36, 631-640. 10.1007/s10875-016-0323-0.

Santoni, G., Farfariello, V., Liberati, S., Morelli, M.B., Nabissi, M., Santoni, M., and Amantini, C. (2013). The role of transient receptor potential vanilloid type-2 ion channels in innate and adaptive immune responses. Frontiers in immunology 4, 34. 10.3389/fimmu.2013.00034.

Schenkel, A.R., Dufour, E.M., Chew, T.W., Sorg, E., and Muller, W.A. (2007). The murine CD99-related molecule CD99-like 2 (CD99L2) is an adhesion molecule involved in the inflammatory response. Cell communication & adhesion 14, 227-237. 10.1080/15419060701755966.

Seo, S., Asai, T., Saito, T., Suzuki, T., Morishita, Y., Nakamoto, T., Ichikawa, M., Yamamoto, G., Kawazu, M., Yamagata, T., et al. (2005). Crk-associated substrate lymphocyte type is required for lymphocyte trafficking and marginal zone B cell maintenance. Journal of immunology 175, 3492-3501. 10.4049/jimmunol.175.6.3492.

Sheikh, F., Dickensheets, H., Pedras-Vasconcelos, J., Ramalingam, T., Helming, L., Gordon, S., and Donnelly, R.P. (2015). The Interleukin-13 Receptor-alpha1 Chain Is Essential for Induction of the Alternative Macrophage Activation Pathway by IL-13 but Not IL-4. Journal of innate immunity 7, 494-505. 10.1159/000376579.
Shema, E., Tirosh, I., Aylon, Y., Huang, J., Ye, C., Moskovits, N., Raver-Shapira, N., Minsky, N., Pirngruber, J., Tarcic, G., et al. (2008). The histone H2B-specific ubiquitin ligase RNF20/hBRE1 acts as a putative tumor suppressor through selective regulation of gene expression. Genes & development 22, 2664-2676. 10.1101/gad.1703008.
Shi, R., Lu, W., Tian, Y., and Wang, B. (2021). Intestinal SEC16B modulates obesity by controlling dietary lipid absorption. bioRxiv. 10.1101/2021.12.07.471468.
Silver, D.P., and Livingston, D.M. (2012). Mechanisms of BRCA1 tumor suppression. Cancer discovery 2, 679-684. 10.1158/2159-8290.CD-12-0221.
Smith, T.J. (2010). Insulin-like growth factor-I regulation of immune function: a potential therapeutic target in autoimmune diseases? Pharmacological reviews 62, 199-236. 10.1124/pr.109.002469.
Strobl, B., Stoiber, D., Sexl, V., and Mueller, M. (2011). Tyrosine kinase 2 (TYK2) in cytokine signalling and host immunity. Frontiers in bioscience 16, 3214-3232. 10.2741/3908.
Tack, J., Deloose, E., Ang, D., Scarpellini, E., Vanuytsel, T., Van Oudenhove, L., and Depoortere, I. (2016). Motilin-induced gastric contractions signal hunger in man. Gut 65, 214-224. 10.1136/gutjnl-2014-308472.
Tsuchiya, M.T.N., Dikow, R.B., Koepfli, K.P., Frandsen, P.B., Rockwood, L.L., and Maldonado, J.E. (2021). Whole-Genome Sequencing of Procyonids Reveals Distinct Demographic Histories in Kinkajou (Potos flavus) and Northern Raccoon (Procyon lotor). Genome Biol Evol 13. 10.1093/gbe/evaa255.
Tuo, L., Xiang, J., Pan, X., Hu, J., Tang, H., Liang, L., Xia, J., Hu, Y., Zhang, W., Huang, A., et al. (2019). PCK1 negatively regulates cell cycle progression and hepatoma cell proliferation via the AMPK/p27(Kip1) axis. Journal of experimental & clinical cancer research : CR 38, 50. 10.1186/s13046-019-1029-y.
Valizadeh, A., Khosravi, A., Zadeh, L.J., and Parizad, E.G. (2015). Role of IL-25 in Immunity. Journal of clinical and diagnostic research : JCDR 9, OE01-04. 10.7860/JCDR/2015/12235.5814.
Wang, K., Xu, R., Snider, A.J., Schrandt, J., Li, Y., Bialkowska, A.B., Li, M., Zhou, J., Hannun, Y.A., Obeid, L.M., et al. (2016). Alkaline ceramidase 3 deficiency aggravates colitis and colitis-associated tumorigenesis in mice by hyperactivating the innate immune system. Cell death & disease 7, e2124. 10.1038/cddis.2016.36.
Wang, Q., Wang, C., Li, N., Liu, X., Ren, W., Wang, Q., and Cao, X. (2018). Condensin Smc4 promotes inflammatory innate immune response by epigenetically enhancing NEMO transcription. Journal of autoimmunity 92, 67-76. 10.1016/j.jaut.2018.05.004.
Yang, L., Liu, C.C., Zheng, H., Kanekiyo, T., Atagi, Y., Jia, L., Wang, D., N'Songo, A., Can, D., Xu, H., et al. (2016). LRP1 modulates the microglial immune response via regulation of JNK and NF-kappaB signaling pathways. Journal of neuroinflammation 13, 304. 10.1186/s12974-016-0772-7.
Yoshinaka, T., Kosako, H., Yoshizumi, T., Furukawa, R., Hirano, Y., Kuge, O., Tamada, T., and Koshiba, T. (2019). Structural Basis of Mitochondrial Scaffolds by Prohibitin Complexes: Insight into a Role of the Coiled-Coil Region. iScience 19, 1065-1078. 10.1016/j.isci.2019.08.056.
Zhan, K., Liu, R., Tong, H., Gao, S., Yang, G., Hossain, A., Li, T., and He, W. (2020). Fetuin B overexpression suppresses proliferation, migration, and invasion in prostate cancer by inhibiting the PI3K/AKT signaling pathway. Biomedicine & pharmacotherapy = Biomedecine & pharmacotherapie 131, 110689. 10.1016/j.biopha.2020.110689.
Zhang, G., Cowled, C., Shi, Z., Huang, Z., Bishop-Lilly, K.A., Fang, X., Wynne, J.W., Xiong, Z., Baker, M.L., and Zhao, W. (2013). Comparative analysis of bat genomes provides insight into the evolution of flight and immunity. Science 339, 456-460.
Zhang, Y.G., Wu, S., Xia, Y., Chen, D., Petrof, E.O., Claud, E.C., Hsu, W., and Sun, J. (2012). Axin1 prevents Salmonella invasiveness and inflammatory response in intestinal epithelial cells. PloS one 7, e34942. 10.1371/journal.pone.0034942.
Zhao, W., Ma, N., Wang, S., Mo, Y., Zhang, Z., Huang, G., Midorikawa, K., Hiraku, Y., Oikawa, S., Murata, M., and Takeuchi, K. (2017). RERG suppresses cell proliferation, migration and angiogenesis through ERK/NF-

kappaB signaling pathway in nasopharyngeal carcinoma. Journal of experimental & clinical cancer research : CR 36, 88. 10.1186/s13046-017-0554-9.
Zhou, H., Li, J., Su, H., Li, J., Lydic, T.A., Young, M.E., and Chen, W. (2022). BSCL2/Seipin deficiency in hearts causes cardiac energy deficit and dysfunction via inducing excessive lipid catabolism. Clinical and translational medicine 12, e736. 10.1002/ctm2.736.
Zhou, H., Xu, C., Lee, H., Yoon, Y., and Chen, W. (2020). Berardinelli–Seip congenital lipodystrophy 2/SEIPIN determines brown adipose tissue maintenance and thermogenic programing. Molecular metabolism 36, 100971. 10.1016/j.molmet.2020.02.014.
Zhu, M., Foreman, D.P., O'Brien, S.A., Jin, Y., and Zhang, W. (2018). Phospholipase D in TCR-Mediated Signaling and T Cell Activation. Journal of immunology 200, 2165-2173. 10.4049/jimmunol.1701291.
